# Inference of brain networks with approximate Bayesian computation – assessing face validity with an example application in Parkinsonism

**DOI:** 10.1016/j.neuroimage.2021.118020

**Published:** 2021-08-01

**Authors:** Timothy O. West, Luc Berthouze, Simon F. Farmer, Hayriye Cagnan, Vladimir Litvak

**Affiliations:** a Nuffield Department of Clinical Neurosciences, Medical Sciences Division, University of Oxford, Oxford OX3 9DU, United Kingdom; b Medical Research Council Brain Network Dynamics Unit, University of Oxford, Oxford OX1 3TH, United Kingdom; c Wellcome Trust Centre for Human Neuroimaging, UCL Institute of Neurology, Queen Square, London WC1N 3BG, United Kingdom; d Centre for Computational Neuroscience and Robotics, University of Sussex, Falmer, United Kingdom; e UCL Great Ormond Street Institute of Child Health, Guildford St., London WC1N 1EH, United Kingdom; f Department of Neurology, National Hospital for Neurology & Neurosurgery, Queen Square, London WC1N 3BG, United Kingdom; g Department of Clinical and Movement Neurosciences, Institute of Neurology, Queen Square, UCL, London WC1N 3BG, United Kingdom

**Keywords:** Inverse modelling, Networks, Brain dynamics, Oscillations, Circuits, Parkinsonism

## Abstract

This paper describes and validates a novel framework using the Approximate Bayesian Computation (ABC) algorithm for parameter estimation and model selection in models of mesoscale brain network activity. We provide a proof of principle, first pass validation of this framework using a set of neural mass models of the cortico-basal ganglia thalamic circuit inverted upon spectral features from experimental, *in vivo* recordings. This optimization scheme relaxes an assumption of fixed-form posteriors (i.e. the Laplace approximation) taken in previous approaches to inverse modelling of spectral features. This enables the exploration of model dynamics beyond that approximated from local linearity assumptions and so fit to explicit, numerical solutions of the underlying non-linear system of equations. In this first paper, we establish a face validation of the optimization procedures in terms of: (i) the ability to approximate posterior densities over parameters that are plausible given the known causes of the data; (ii) the ability of the model comparison procedures to yield posterior model probabilities that can identify the model structure known to generate the data; and (iii) the robustness of these procedures to local minima in the face of different starting conditions. Finally, as an illustrative application we show (iv) that model comparison can yield plausible conclusions given the known neurobiology of the cortico-basal ganglia-thalamic circuit in Parkinsonism. These results lay the groundwork for future studies utilizing highly nonlinear or brittle models that can explain time dependant dynamics, such as oscillatory bursts, in terms of the underlying neural circuits.

## 1 Introduction

Models of mesoscale brain activity (Vogels et al., 2005; Deco et al., 2015; Breakspear, 2017) provide ways to understand how interactions between: (a) the *function* of neurons (dictated by their intrinsic biophysical properties); and (b) the *structure* of the synaptic network that connects them, can modulate neural communication. Typically, the integration of activity across spatially distributed networks has been estimated using the tools of functional connectivity (i.e. determining the statistical dependencies between brain activity; Friston, 2011). However, these approaches are descriptive and so are unable to explore the causes of correlated neural activity that can be explained through changes in either structure, function, or a combination of both.

By building generative models of neural circuit dynamics and then *inverting* them from data, it is possible to gain insight into the mechanisms underlying the rich spatiotemporal patterning of brain activity (Horwitz et al., 2000). Inverse modelling not only allows for the prediction of an individual model's parameters, but also the comparison of models, so allowing different hypotheses to be evaluated given some data (Jaqaman and Danuser, 2006). In its simplest form, combining *a priori* knowledge alongside hand tuning of unknown parameters has led to a number of sophisticated models (Traub et al., 1991; De Schutter and Bower, 1994). This approach can be formalized, using algorithmic schemes for parameter estimation (Rowe et al., 2004; Wendling et al., 2009). More recently, Bayesian optimization schemes provide a principled way of including prior knowledge of a system when computing an inverse model (Moran et al., 2009; Hadida et al., 2018; Hashemi et al., 2018). These approaches can estimate the posterior distribution over model parameters (i.e. parameter estimation) as well as over a space of models (i.e. model evidence).

Many approaches make assumptions to render a model amenable to a particular optimization scheme. For instance, in Dynamic Causal Modelling (DCM; Friston et al., 2012), the optimization algorithm (variational Bayes) makes an assumption of fixed form posteriors (the Laplace approximation). This simplifies the optimization problem by reducing the description of the approximate posterior density to its first two moments but precludes the examination of highly nonlinear or stochastic models, where this is likely to be violated by the existence of multimodal posteriors (Daunizeau et al., 2009; Sengupta et al., 2016). To help ensure this assumption is met, a model can be linearized and its behaviour approximated by computing its transfer function around a fixed point (Valdes et al., 1999; Robinson et al., 2001; Friston et al., 2012). This helps to ensure that posterior densities conform to a multivariate normal but can limit the model dynamics that may be explored (although see discussion for existing approaches to this problem). Importantly, highly nonlinear or stochastic dynamics are thought to underpin significant features observed in the functional organization of brain activity, such as the transitions between resting-states (Deco et al., 2009, 2011) or the transient bursting of synchronous activity (e.g. Palmigiano et al., 2017). In this work we describe a framework for the inverse modelling of large-scale brain dynamics that avoids: (a) appeals to the Laplace approximation; and (b) approximating model dynamics from local linear behaviour.

To these ends, we set up a framework using the Approximate Bayesian Computation algorithm (ABC; Beaumont et al., 2002) that provides a method of “simulation based” inference (Cranmer et al., 2020) and is well suited to complex models which have a large state and/or parameter space, exhibit stochastic or highly nonlinear dynamics, or require numerically expensive integration schemes to solve. This method has been successfully employed and validated across a number of models in systems biology (Excoffier, 2009; Toni and Stumpf, 2009; Turner and Sederberg, 2012; Liepe et al., 2014), but is yet to see wide usage in neuroscience. Usefully, the scheme allows models to be inverted upon hypothetically any summary statistic of neural recordings such as spectra, spike density, or measures of connectivity (although see discussion for a description of the risk of “insufficiency” in these features). Specifically, we use a variant of ABC called sequential-Monte Carlo ABC (ABC-SMC; Toni et al., 2009).

We aim to provide a first-pass evaluation of the face validity of the proposed framework. To do this, we build a set of examples using models of Parkinsonian circuit dynamics. These examples are derived from a previously reported model of the cortical-basal ganglia-thalamic circuit (van Wijk et al., 2018) and constrained using data from an experimental, rodent model of Parkinsonism (West et al., 2018). We use a reduced set of data features (the magnitude of the power spectra and directed functional connectivity) that can be derived from the full complex cross-spectra, and simplify the estimation of the observation model (see methods). Note that we retain relatively simple, time-averaged data features, as well as a well-established generative model, in order focus our examination upon the validity of the optimization scheme itself. This then paves the way for further validations of this method in terms of more complicated models and data (see discussion).

Specifically, we first examine the properties of the inversion scheme, investigating parameter estimates and convergence. We then take a similar approach to that used previously in the validation of methods such as DCM by first testing the so-called *face* validity (Moran et al., 2009) through examination of (a) the ability of the parameter estimation procedure to yield plausible posterior distributions over parameters given those known to generate the data; (b) the robustness of the parameter estimation method to the existence of local minima in the face of multiple realizations of the same data and different starting conditions; and (c) the ability of the model comparison procedures to recover plausible model architectures given that known to generate the data. Finally, we demonstrate that the scheme can yield neurobiologically plausible conclusions given the structure of the circuits known to underly oscillatory dynamics in Parkinsonism.

## 2 Methods

### 2.1 Overview of sequential Monte Carlo approximate Bayesian computation for inverse modelling of neural data

**Fig. 1:**
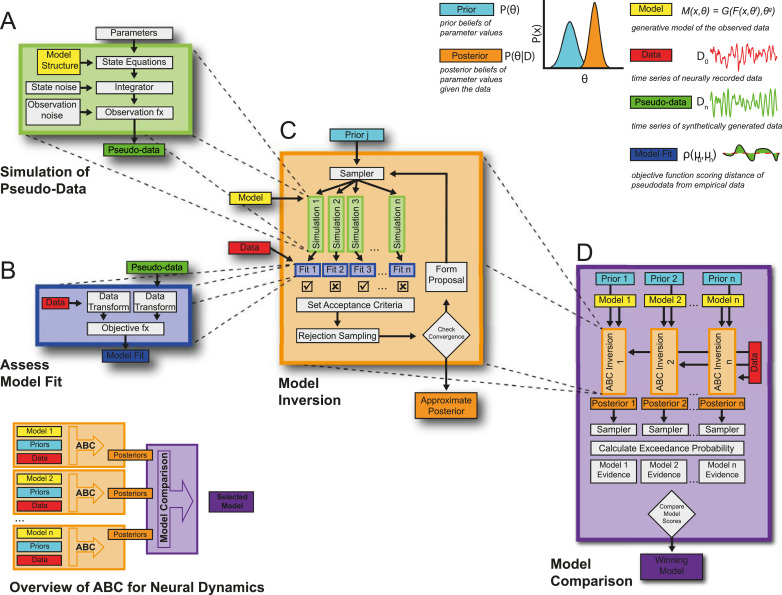
**Framework for application of Approximate Bayesian Computation for simulation-based inference of brain network dynamics. (Inset)** This schematic gives an overview of the framework described in this paper. Individual generative models are specified as a set of state equations (yellow boxes) and prior distribution over parameters (turquoise boxes) that will be used to approximate the posterior density over the parameters (orange boxes) for the system generating the observed data (red boxes) with varying degrees of fit (dark blue boxes) using ABC. The approximate posterior distribution can then be used to compare models and decide on a winning model or family of models (purple box). **(A) Generation of pseudo-data** by integrating the state equations parameterized by a sample drawn from the prior or proposal distributions. Models can incorporate stochastic innovations as well as a separate observation model to produce samples of pseudo-data (green boxes). **(B) Pseudo-data is compared against the real data** using a common data transform that provides a summary statistic of the time series data (i.e. spectra and directed functional connectivity). The simulated and empirical data are then compared by computing the objective function that can be used to score the model fit (blue boxes). **(C) ABC sequentially repeats the processes in boxes A and B** by iteratively updating a proposal distribution formed from accepted samples. Samples are rejected depending on an adaptive threshold on the objective scores aiming to reduce the distance between summary statistics of the data and pseudo-data. This process iterates until the convergence criterion is met and the proposal distribution is taken as an approximation to the posterior distribution. **(D) By repeating the ABC process in box (C) over multiple models**, the approximate posteriors can be used to evaluate the model probabilities. This process samples from the posterior many times to compute the probability of each model exceeding the median accuracy of all models tested. This acceptance probability can then be used to compare the model's ability to accurately fit the data and select the best candidate model given the data. (For interpretation of the references to colour in this figure legend, the reader is referred to the web version of this article.)

We present an overview of the framework using ABC-SMC and its adaptations for applications to large scale neural models in Fig. 1. The algorithm takes a form in which several processes are repeated multiple times within their parent process (Fig. 1; inset). The scheme is contingent on simulation of pseudo-data by a generative forward model – a description of the neural dynamics - (Fig. 1A; green box) given a set of proposal parameters sampled from a prior (multivariate Gaussian) distribution (Fig. 1C; turquoise box). This pseudo-data can then be compared against the empirical data by first using a common data transform (i.e. a summary statistic of the data) and then assessing their similarity by computing the objective function (goodness-of-fit) (Fig. 1B; blue box). This model fit provides a measure by which parameter samples are either rejected or carried forward depending on a threshold on the goodness-of-fit, in order to generate the next proposal distribution in the sequence. When the process in Fig. 1C is iterated with a shrinking tolerance schedule, ABC can be used to approximate the posterior parameter distribution at convergence (Fig. 1C; orange box). Finally, if the process described above is repeated over several competing models then the approximate posterior distribution may be used to assess each model's fitness via model comparison (Fig. 1D; purple box). The exact details of each process outlined in the figure are given in the text below. The existing methods that form the basis for this work are outlined in appendix I.

### 2.2 Forward model for generation of neural pseudo-data

The fitting algorithm is based upon sampling from a sequence of proposal distributions over parameters to generate realizations of the generative process that we refer to as pseudo-data (Fig. 1A; green box). A model M is specified by the state equations of the dynamical systemF and observation model G:

(1) $$M\left(x,\theta \right)=G\left(F\left(x,\theta^{F}\right),\theta^{G}\right).$$

The equations of the model *F* describe the evolution of states *x* with parameters $\theta^{F}$. This model describes the underlying neuronal dynamics that give rise to the evolution of the states. The observation model *G* describes the effects upon the signals that are introduced by processes such as experimental acquisition or recording and is parameterized by $\theta^{G}$. The observation model can account for confounds introduced beyond that of the generative model of the data such as changes in signal-to-noise ratio (SNR), crosstalk, or other distortions of the underlying generative process. In the examples provided here, we use a simple observation model that comprises a model of sensor noise in which the gain on additive noise remains a free parameter to estimate differences in the SNR between signals. We use Gaussian white noise and assume identity covariance between the sensors. In this example, we avoided estimation of the lead field parameters (Kiebel et al., 2006) by our choice of summary statistic describing the interaction between sources (see section Directed Functional Connectivity).

In general, model *M* could describe any dynamical system describing the time evolution of neural data such as spiking networks, conductance models, or phenomenological models (e.g. phase oscillators, Markov chains). In this paper we use coupled neural mass equations (Jansen and Rit, 1995; David and Friston, 2003) to model population activity of the cortico-basal-ganglia-thalamic circuit, of which the biological and theoretical basis has been previously described (Moran et al., 2011; Marreiros et al., 2013; van Wijk et al., 2018). The original equations were adapted to explicitly incorporate stochastic inputs and finite transmission delays. This yielded a system of stochastic delay differential equations that could be solved using the Euler-Maruyama method. For details of the modelling, as well as details of the integration of the state equations please see Supplementary Information I which gives details of the model formulation, state equations, and numerical solver.

Parameters of both the generative and observational model can either be fixed or variable. In the case of variable parameters (parameters to be fit), a prior density encoding *a priori* beliefs about the values that the parameters take must be specified. This is encoded through the mean and variance for each parameter, with the variance encoding the inverse-precision of a prior belief. In this way fixed parameters can be thought as being known with complete confidence. Note we assume identity covariance of the priors. We take the majority of prior values for parameters of the cortico-basal-ganglia-thalamic network from (van Wijk et al., 2018) but set some delays and connection strengths given updated knowledge in the literature. A table of parameters can be found in Supplementary Information II.

For results in Sections 3.1 through to the first part of Section 3.3 we use a reduction of the full model comprising the reciprocally coupled STN/GPe. This model can be again divided into separate models (for the purposes of performing face validation and example model comparison) by constraining priors on the connectivity between the STN and GPe. The later part of Section 3.3 and Section 3.4 uses a wider model space comprising a set of systematic variations upon the full model (see Fig. 5).

### 2.3 Model inversion with sequential Monte Carlo ABC

#### 2.3.1 Algorithm overview

In order to estimate the parameters of the model *M*, given the information provided by the empirical recordings we use an algorithm based upon ABC-SMC (Toni et al., 2009; Del Moral et al., 2012). ABC is a “likelihood free” algorithm (Marin et al., 2012). Most generally, the algorithm forms a sample of draws taken from a prior distribution and then goes on to estimate an intermediate sequence of proposal distributions via iterative rejection of the parameter draws. Given a suitable shrinking tolerance schedule, the simulated pseudo-data (generated from the sample-parameterized forward model) and the empirical data should converge as the proposal distribution approaches the true posterior.

The ABC algorithm is illustrated in Fig. 1C (orange box) and follows the procedure below. Probability densities are given by P(·); parameters are indicated by θ; models by *M*; data by *D;* and distances by ρ. Samples are indicated by hat notation (i.e. $\hat{\cdot }$); subscripts indicate the sample number; proposal distributions are indicated by an asterisk (i.e. $P{\left(\cdot \right)}^{*}$); and subscripts equal to zero denote belonging to the empirical data (i.e. $\mu_{1}$ and $\mu_{0}$are summary statistics of sample 1 and of the empirical data respectively).

**Table:** 

1 *Specify prior distribution of parameters,* P(θ) *of the model M.*	*(model prior)*
2 *Randomly sample* N *times from the prior to yield samples* ${\hat{\theta }}_{n}$*.*	*(sampler)*
3 *Simulate pseudo-data* ${\hat{D}}_{n}$ *~* $M(x_{n},{\hat{\theta }}_{n}$*).*	*(simulation of joint distribution)*
4 *Compute summary statistic of* $\mu_{n}$ *of pseudo-data* ${\hat{D}}_{n}$*.*	*(data transform)*
5 *Compute distance* ρ *of* $\mu_{n}$ *from* $\mu_{0}$*.*	*(assess model fit)*
6 *Reject* ${\hat{\theta }}_{n}$ *if distance* $\rho \left(\mu_{n},\mu_{0}\right)\geq \epsilon_{q}.$	*(rejection sampling)*
7 *Form proposal distribution,* $P^{*}\left(\theta |D_{0}\right)$*, from the accepted parameter samples.*	*(form proposal)*
8 *Iterate for* q=1,…,Q*, setting* $P\left(\theta \right)=\;P^{*}\left(\theta |D_{0}\right)$*, and shrinking the distance threshold.*	*(adaptive tolerance schedule)*
9 *At convergence criteria, accept proposal distribution as posterior:* $P\left(\theta |D_{0}\right)≅P^{*}\left(\theta |D_{0}\right)$*,*	*(estimation of approximate posterior)*

To avoid sample wastage across iterations, we store the samples from step (2) and their resulting distance from the data (step 5) across the *Q* iterations such that they are propagated through a sequence of intermediate distributions. In this way, at step (7) the updated proposal distribution comprises samples from both current and past draws selected on the basis of a threshold calculated over the current draw.

We estimate the “distance” or error of the pseudo-data from the real data using the pooled mean squared error (MSE_pooled_) as an objective function $\rho (\mu_{n},\mu_{0})$ (see supplementary information II for equation). Setting a shrinking distance threshold ϵ ensures that the posterior estimates converge upon solutions that most accurately reproduce the summary statistics of the observed data (Del Moral et al., 2012). With non-negligible $\epsilon_{Q}$, the algorithm samples from an approximate posterior distribution $P(\theta |\rho \left(\mu_{n},\mu_{0}\right)<\epsilon_{Q})\;$rather than the true posterior P(θ|D) when $\epsilon_{Q}\to 0$. Thus the upper bound on the error of parameter estimates is therefore determined by how far $\epsilon_{Q}\;$is from zero (Dean et al., 2014).

#### 2.3.2 Adaptive tolerance schedule

To facilitate incremental sampling from a sequence of increasingly constrained target distributions we set an adaptive tolerance schedule. This is specified by determining a predicted gradient for the average distance of the next set of samples:

(2) $$\epsilon_{q+1}=\epsilon_{q}+\Delta \epsilon_{q}$$

where the expected change in the distance of the new samples $\Delta \epsilon_{q}$ is given by:

(3) $$\Delta \epsilon_{q}=\{\begin{array}{c} \epsilon_{q}-\epsilon_{q-1},\;N_{accept}\geq \gamma \\ \epsilon_{q}^{*}-\epsilon_{q-1},\;N_{accept}<\gamma \end{array}$$

where $N_{accept}$ is the number of accepted samples and γ is a minimum criterion on the accepted sample size to carry forward the tolerance shrinkage at its current gradient. If $N_{accept}<\gamma$ then this gradient is assumed to be too steep and the expected gradient is recalculated using a modified tolerance $\epsilon_{q}^{*}$ that is computed using the median distance ρ between the sample pseudo-data from that real (i.e. $\epsilon_{q}^{*}=\;\tilde{\rho }$, where ~ indicates the median). Thus γ parameterizes the coarseness of the optimization. If γ is very large (e.g. >99% of *N*) then the algorithm will converge slowly but accurately, whereas if γ is very small (e.g. 1% of *N*) the algorithm will be inaccurate and biased. We set γ to be the two times the estimated rank of the parameter covariance matrix i.e. rank(Σ) (for details of estimation see Section 2.3.3).

#### 2.3.3 Formation of proposal distributions

Following rejection sampling, the proposal density $P^{*}\left(\theta |D_{0}\right)$ is formed from the accepted parameters sets. We use a density approximation to the marginals and a copula for the joint similar to that described in Li et al. (2017). We take the initial draw of samples from the prior formed with a multivariate normal:

(4) $$P^{*}\left(\theta \right)=N\left(\mu ,\Sigma \right)$$

where μ is a vector of the prior expectations and Σ their covariances. In subsequent iterations whereby a minimum sample is accumulated, we use nonparametric estimation of the marginal densities over each parameter using a non-parametric kernel density estimator (Silverman, 2003). This approach allows for free-form approximation to probability densities (e.g. multimodal or long-tailed distributions). This flexibility allows for sampling across multiple possible maxima at once, particularly at intermediate stages of the optimization. The bandwidth (determining the smoothness) of the kernel density estimator is optimized using a log-likelihood, cross-validation approach (Bowman, 1984).

We then form the multivariate proposal distribution using the t-copula (Nelsen, 1999). Copula theory provides a mathematically convenient way of creating the joint probability distribution whilst preserving the original marginal distributions. Data are transformed to the copula scale (unit-square) using the kernel density estimator of the cumulative distribution function of each parameter and then transformed to the joint space with the t-copula.

The copula estimation of the correlation structure of the parameter distributions acts to effectively reduce the dimensionality of the problem by binding correlated parameters into modes. The effective rank of the posterior parameter space (used in the computation of the adaptive tolerance schedule and reported in the results as a post-hoc assessment of parameter learning) can be estimated by taking the eigenvalues of the parameter covariance matrix and normalizing the coefficients by their sum. Using a cumulative sum of the ordered coefficients we can then determine the number of modes that can explain 95% of the variance of the parameter samples.

### 2.4 Model comparison

In the process of model-based inference, hypotheses may be compared in their capacity to explain the observed data. Models fit with ABC can be formally compared using either “joint-space” or “marginal likelihood” based approaches (Grelaud et al., 2009; Toni and Stumpf, 2009). Here we use the latter approach which estimates the marginal likelihood (model evidence) for each *j^th^* model:

(5) $$P\left(D_{0}|M^{j}\right)≅\frac{\#\;\rho \left(\mu_{n},\mu_{0}\right)\leq \hat{\epsilon }}{N}$$

where $\hat{\epsilon }$ is a threshold on the distance metric ρ that is suitably small to give an acceptable fit on the data and is common across models. We refer to the outcome of Eq. (5) as the acceptance rate of a particular model. For derivation of the ABC-SMC approximation to the marginal likelihood, please see Toni and Stumpf (2009). In practice we set $\epsilon^{*}$ to be the median of the distances across of all sets of models. The marginal posterior probability of a model is then given by combining marginal model likelihoods and prior model probability $P(M^{j})$ and then normalizing across the space of *J* models:

(6) $$P\left(M^{j}D_{0}\right)=\frac{P\left(D_{0}|M^{j}\right)P\left(M^{j}\right)}{\sum_{i=1}^{J}P\left(D_{0}|M^{i}\right)P\left(M^{i}\right)}$$

In all cases described, we assume that prior model probabilities are uniform.

We provide a post-hoc estimation of model complexity terms of the divergence of the posterior from the prior (Friston et al., 2007; Penny, 2012). Specifically, we estimate the Kullback-Lieber divergence D_KL_ of the posterior density $P(\theta |D_{0})$ from the prior density P(θ)over *F* discretized bins of the density:

(8) $$D_{KL}\left(P\left(\theta |D_{0}\right)∥P\left(\theta \right)\right)=-\sum_{i=1}^{F}P\left(\theta \right)\;log\left(\frac{P\left(\theta \right)\left(i\right)}{P\left(\theta |D_{0}\right)\left(i\right)}\right)$$

This is a simplification of the full multivariate divergence and ignores the dependencies between variables encoded in the posterior covariance. We use the full multivariate divergence (given in supplementary information IV) that uses a multivariate Gaussian approximation to the ABC estimated posterior density (taking the mean and covariance of *N* samples from the posterior). The D_KL_ can then be used for a post-hoc discrimination of model performance. To do this we build a complexity-adjusted goodness-of-fit heuristic (accuracy-complexity score; ACS) similar in form to an information criterion such as the Bayesian Information Criterion, but refined to consider posterior divergence as a measure of complexity, rather than the absolute number of parameters:

(9) $$\mathrm{AC}S^{j}=\;-\log_{10}\left(P\left(M^{j}|D_{0}\right)\right)-\log_{10}\left(\frac{J\;D_{\mathrm{KL}}^{j}}{\sum_{i=1}^{J}D_{\mathrm{KL}}^{i}}\right)$$

where $D_{KL}^{j}$ is the divergence of posteriors from priors for the *j^th^* model normalized by the sum of divergences across the whole model space. Models that contribute exactly 1/J of the summed divergence have zero complexity penalty. Please note that the ACS does not provide the objective function for optimization (which is the pooled MSE), but rather a heuristic for post-hoc discrimination of models via the addition of an Occam factor to account for model parsimony (MacKay, 2003).

### 2.5 Empirical data: recordings from Parkinsonian rats

Summary statistics are computed from empirical data and then used to fit the generative forward model. In the example implementation used in this paper we use multisite basal ganglia and single site cerebral cortex recordings in rats (*n* = 9) that have undergone a 6-hydroxydopamine (6-OHDA) induced dopamine depletion of the midbrain, a lesion model of the degeneration associated with Parkinsonism in humans (Magill et al., 2004, 2006). The animals were implanted with two electrodes to measure local field potentials (LFP) from multiple structures in the basal ganglia: dorsal striatum (STR), external segment of the globus pallidus (GPe), and the subthalamic nucleus (STN). Additionally electrocorticography was measured over area M2 of the motor cortex, a homologue of the Supplementary Motor Area (SMA) in humans (Paxinos and Watson, 2007). Animals were recorded under isoflurane anaesthesia and during periods of “cortical-activation” induced by a hind-paw pinch (Steriade, 2000). The details of the experimental procedures were previously published (Magill et al., 2004, 2006). Experimental procedures were performed on adult male Sprague Dawley rats (Charles River) and were conducted in accordance with the Animals (Scientific Procedures) Act, 1986 (UK), and with Society for Neuroscience Policies on the Use of Animals in Neuroscience Research. anaesthesia was induced with 4% v/v isoflurane (Isoflo; Schering-Plough) in O2 and maintained with urethane (1.3 g/kg, i.p.; ethyl carbamate, Sigma), and supplemental doses of ketamine (30 mg/kg, i.p.; Ketaset; Willows Francis) and xylazine (3 mg/kg, i.p.; Rompun, Bayer).

Pre-processing of time series data (LFP and ECoG) was done as follows: data were 1) truncated to remove 1 second (avoid filter artefacts); 2) mean corrected; 3) band-passed filtered 4–100 Hz with a finite impulse response, two-pass (zero-lag) with optimal filter order; 4) data were split into 1 second epochs with each epoch subjected to a Z-score threshold criterion such that epochs with high amplitude artefacts were removed.

### 2.6 Computation of summary statistics

We derive a set of summary statistics from signal analyses of the experimental and simulated time series. These statistics transform both the data and pseudo-data into the same feature space such that they can be directly compared (Fig. 1B; blue box). It is important to note that the summary statistic is vital in determining the outcome of the inverse modelling with ABC (Beaumont et al., 2002; and see discission). The set of statistics must effectively encode all phenomena of the original data that the experimenter wishes to be modelled.

#### 2.6.1 Frequency spectra

We use the autospectra to constrain the oscillatory activity of each neural mass. Auto-spectral analyses were made using the averaged periodogram method across 1 second epochs and using a Hanning taper to reduce the effects of spectral leakage. Frequencies between 49 and 51 Hz were removed so that there was no contribution from 50 Hz line noise. 1/f background noise was removed by first performing a linear regression on the log-log spectra (at 4–48 Hz) and then subtracting the linear component from the spectra (Le Van Quyen et al., 2003; Nikulin and Brismar, 2006). Note that only empirical data underwent removal of the 1/f background. This ensured that the inversion scheme was focused upon fitting the spectral peaks in the data and not the profile of 1/f background noise. To simplify observation modelling of differences in experimental recording gains between sites, all spectra were normalized by dividing through by their summed power at 4–48 Hz.

#### 2.6.2 Directed functional connectivity

To quantify interactions between populations, we use non-parametric directionality (NPD; Halliday 2015), a directed functional connectivity metric which describes frequency-resolved, time-lagged correlations between time series. The NPD was chosen as it makes it possible to remove the zero-lag component of coherence and so interactions between signals are not corrupted by signal mixing/volume conduction, of which was predominant in the empirical data used here (see West et al., 2018). Thus, using NPD simplifies the observation problem by removing the need to estimate mixing terms or a lead field.

Estimates of NPD were obtained using the Neurospec toolbox (http://www.neurospec.org/). This analysis combines Minimum Mean Square Error (MMSE) pre-whitening with forward and reverse Fourier transforms to decompose coherence estimates at each frequency into three components: forward, reverse and zero lag. These components are defined according to the corresponding time lags in the cross-correlation function derived from the MMSE pre-whitened cross-spectrum. This approach allows the decomposition of the signal into distinct forward and reverse components of coherence separate from the zero-lag (or instantaneous) component of coherence which can reflect volume conduction. The method uses temporal precedence to determine directionality. For a detailed formulation of the method see Halliday (2015); and for its validation see West et al. (2020b). We ignored the instantaneous component of the NPD and use only the forward and reverse components for all further analyses. Note that NPD accounts for the relative phase between activities by segregating the contribution to the cross-spectrum into either leading (forward) or lagging (reverse) components.

#### 2.6.3 Data pooling and smoothing

In all procedures using empirical data to constrain models, we used the group-averaged statistics computed from recordings from a group of unilaterally 6-OHDA lesioned animals. As a final processing step, both the autospectra and NPD were smoothed to remove noise such that fitting was focused on the dominant peaks of the features (Rowe et al., 2004). This was achieved by convolving spectra with a 4 Hz wide Gaussian kernel. Empirical and simulated data were transformed identically to produce equivalent autospectra and NPD. We assume smoothness of the spectral features as a way to separate actual features from noise. Spectral estimates in neuroscience (and in general) become increasingly smooth with large sample sizes (with error decreasing relative to √n), thus the smoothing can be considered a correction for finite time spectral estimates.

#### 2.6.4 Software availability

All analyses and procedures were written in MATLAB (Mathworks, Natick, MA). The toolbox is publicly available and maintained as a Github repository (https://github.com/twestWTCN/ABCNeuralModellingToolbox.git). For a list of external dependencies and their authors see appendix I. The procedures used for constructing the figures in this paper can be run using the ‘West2021_Neuroimage_Figures.m’ script. Please see appendix IV for a short guide to the repository and its key scripts.

### 2.7 Validation of ABC procedures for parameter inference and model identification

#### 2.7.1 Testing the face validity of the model inversion procedures

To test whether the ABC estimator will: a) yield parameter estimates that are unique to the data from which they have been optimized; and b) yield consistent estimation of parameters across multiple instances, we performed two procedures of eight multi-starts using two separate datasets. The datasets were created by first defining two forward models with different parameter sets: (1) the MAP estimate of a reciprocally coupled STN/GPe after fitting to the empirical data and (2) the same model but with each parameter log scaling factors randomly adjusted by ±1. These models were then used to generate synthetic datasets by simulating 256 s of data (with separate realizations for each multi-start). We could then track the error of parameter estimates from the known parameters of the original forward model to examine the accuracy of the inference.

When testing point (a), that parameter estimates are unique to the data from which they are fitted - we performed a eight-fold cross-validation procedure in which we used a one-sample Hotelling procedure to test for significant difference of each fold's mean from that of the left-out sample. We report the probability of the folds that yielded a significant test, with high probability indicating that the left-out MAP estimates are likely to deviate from the rest of the fold. In this way we can identify the probability of an ABC initialization yielding a non-consistent sample. Secondly, we test (b), that MAP estimates are unique to the data on which they have been fitted- using the Szekely and Rizzo energy test (Aslan and Zech, 2005) between the samples from data A and B, with the null-hypothesis that the multi-start samples derived from different data arise from the same distribution. Finally, we use a Multivariate Analysis of Variance (MANOVA) procedure to test for difference in means between the two multivariate samples.

#### 2.7.2 Testing the face validity of the model comparison procedures

To test the face validity of the model comparison framework, we constructed a confusion matrix, an approach commonly used in machine learning to examine classification accuracy. Three different models of the STN/GPe network were fit to the empirical data and then using the fitted parameters three synthetic data sets were simulated. We chose a model with reciprocal connectivity: (1) STN ↔ GPe; and then two models in which one connection was predominant: (2) STN → GPe and (3) GPe → STN. The three models (with the original priors) were then fitted back onto the synthetic data. Model comparison was then performed to see whether it could correctly identify the original mode that had generated the data. The model comparison outcomes (accuracy of the fit; D_KL_ of posteriors from priors; and the combined ACS measure) were then plotted in a 3 × 3 matrix of data versus models. In the case of valid model selection, the best fitting models should lay on the diagonal of the confusion matrix. We also performed a second analysis in which more complex models were fitted (incorporating between 4 and 6 sources). Specifically, we used models M1.1, M2.2, and 5.2 described in detail in the next section (2.7.3).

#### 2.7.3 Testing the scalability of the framework with application to the full model space

In order to demonstrate the scalability of the optimization and model comparison framework, we used the space of 12 models described below. We individually fitted the models and then performed a (marginal likelihood-based; see methods) model comparison to select the best of the candidate models. A set of null models were included which are anatomically implausible. If model selection performed correctly, then it is expected that these models would perform poorly.

To investigate the importance of known anatomical pathways in reconstructing the observed steady state statistics of the empirical local field potentials (i.e. autospectra and NPD), we considered a set of competing models. Specifically, we looked at the role of five pathways and their interactions: the cortico-striatal indirect; the cortico-striatal direct; the cortico-subthalamic hyperdirect; thalamocortical relay; and the subthalamic-pallidal feedback. In total we tested 6 families of models (presented later in the results section- Fig. 5):

1 + indirect.
2 + indirect / + hyperdirect.
3 – indirect / + hyperdirect.
4 + indirect / + direct / – hyperdirect / + thalamocortical.
5 + indirect / + direct / + hyperdirect / + thalamocortical.
6 - indirect / + direct / + hyperdirect / + thalamocortical.

We considered these six families and further divided them into two sub-families that do or do not include the subthalamopallidal (STN → GPe) feedback connection. Family (1) investigates whether the indirect pathway alone can explain the pattern of observed spectra and functional connectivity. In the case of family (2), previous work has highlighted the importance of hyper-direct connections in the functional connectivity (Jahfari et al., 2011; Nambu et al., 2015), yet anatomical research has shown dopamine to weaken the density of synaptic projections (Chu et al., 2017). Thus, family (2) provides an ideal set of models to examine the nonlinear mapping of anatomical to functional connectivity described in the introduction of this paper. Families (3) and (6) represent *null* models in which the indirect pathway is excluded and are used as implausible models to test whether the model comparison procedure yields valid results given the known neurobiology. This is because the indirect pathway is thought to be vital in explaining anetwork activity following the dopamine depletion associated with PD (Alexander et al., 1986; Albin et al., 1989; Bolam et al., 2000). The functional role of the thalamocortical subnetwork is relatively unknown (but see recent work: Reis et al., 2019) and so families (4) and (5) provide an examination of whether the addition of the thalamic relay can better explain the empirical data. The second level of families (i.e. x.1–2) investigates whether the reciprocal network formed by the STN and GPe is required to explain observed patterns of connectivity in the data. This network has been the subject of much study and is hypothesized to play an important role in the generation and/or amplification of pathological beta rhythms (Plenz and Kital, 1999; Bevan et al., 2002; Cruz et al., 2011).

## 3 Results

### 3.1 Properties of fitting procedure and convergence when applied to a simple model of the pallido-subthalamic subcircuit

**Fig. 2:**
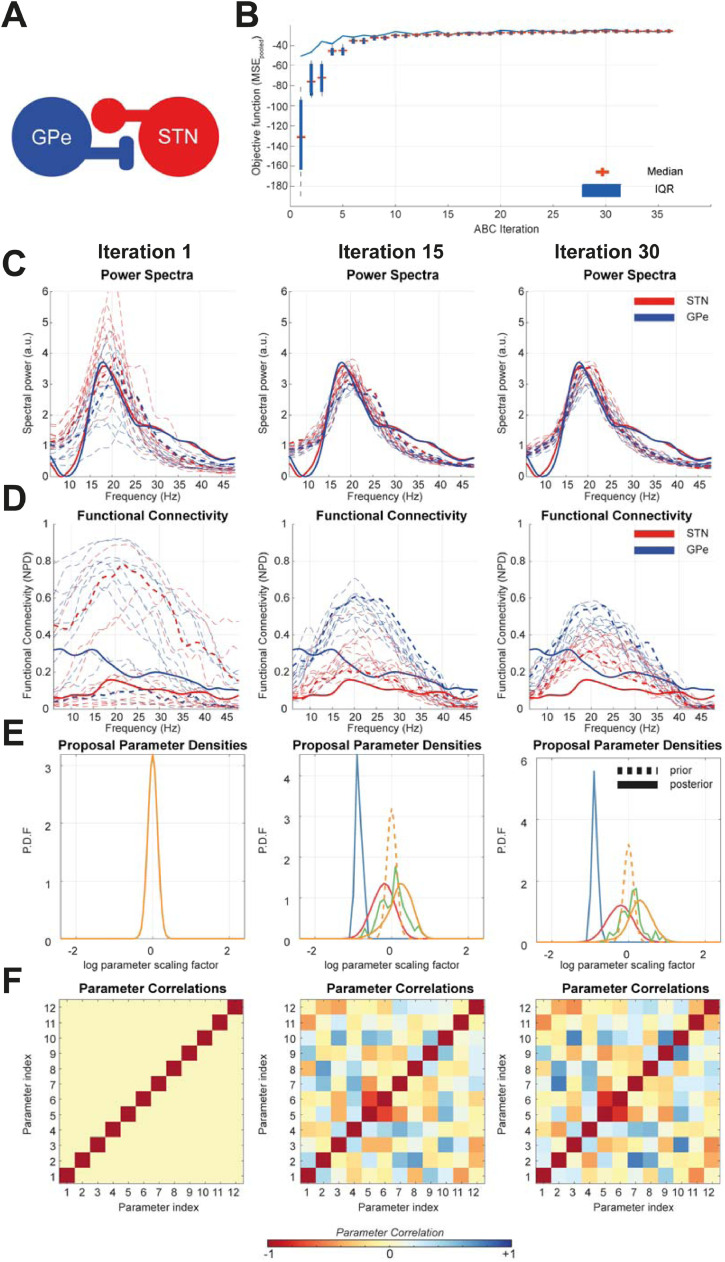
**Examining the convergence of ABC optimization upon summary statistics from recordings of the STN and GPe in Parkinsonian rats –** Parameters of neuronal state space models were optimized using the ABC method detailed in the text. Snapshots of the optimization are taken at the 1st, 15th, and 30th iteration at which the optimization converges. **(A)** Schematic of the STN/GPe neural mass model. **(B)** The iteration history of the ABC algorithm is presented as a sequence of box plots indicating the distribution of fits (MSE_pooled_) at each sampling step, with mean and interquartile range indicated by individual crosses and boxes. **(C)** Power spectra of the empirical data (bold) and simulated data (dashed) are shown. The best fitting parameter sample for each iteration is given by the bold dashed line. **(D)** Similarly, the functional connectivity (non-parametric directionality; NPD) is shown in red and blue with the same line coding. **(E)** Examples of the prior (dashed) and proposal (bold) marginal distributions for a selection of five parameters are shown (note some priors have identical specifications and so overlap). It is seen that over iterations the proposal and posterior deviate from the prior as the latent parameter densities are estimated. **(F)** Correlation matrices from copula estimation of joint densities over parameters. Colour bar at bottom indicates the correlation coefficient. Correlated modes appear between parameters as optimization progresses. (For interpretation of the references to colour in this figure legend, the reader is referred to the web version of this article.)

Fig. 2 shows the results of an example model inversion demonstrating how the ABC algorithm iteratively converges to yield an approximation to the summary statistics of the empirical data. This example uses a simple model comprising the reciprocally connected subthalamic nucleus (STN) and external segment of the globus-pallidus (GPe) shown in Fig. 2A. The autospectra and directed functional connectivity were fit to the group averaged results originally reported in West et al. (2018) which described an analysis of local field potentials recorded from a rodent model of Parkinsonism (see methods for experimental details). By tracking the value of the objective function (i.e. the MSE_pooled_) over multiple iterations (Fig. 2B) we demonstrate a fast-rising initial trajectory in the first 15 iterations that eventually plateaus towards convergence. In Fig. 2C and D the simulated features (autospectra and NPD respectively) gradually move closer to the empirically estimated features with each iteration of the algorithm.

The evolution of the proposed marginal densities (Fig. 2E) demonstrates that over the optimization, parameter means, and variances deviate significantly from the prior. Estimation of some parameters are better informed by the data than for others, as indicated by the different precision of the proposal densities. Additionally, learnt multivariate structure in the joint parameter densities is apparent in the parameter correlation matrices (see methods; Fig. 2F). The evolution of these matrices shows the emergence of distinct correlated modes. These modes reduce the dimensionality of the optimization problem: by estimating the number of significant principal components of the parameters (see methods) we find that optimized models show a reduction of 50–70% from that of the prior. Note however, that due to the identity covariance of the prior, increased correlation in parameters entails an increase the complexity penalty in the ACS metric used to discriminate between models (see Methods and below).

### 3.2 Testing the internal consistency of data dependant estimation of ABC optimized posteriors using a multi-start procedure

**Fig. 3:**
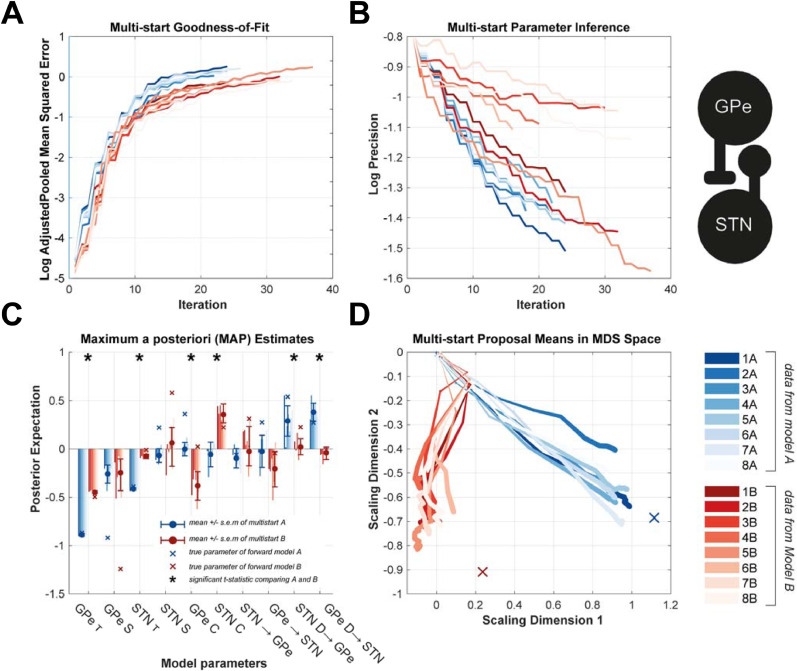
**Multi-start analysis to test face validity of the ABC-based estimation of model parameters by demonstrating consistency of estimation and the data specificity of parameter estimates.** A two-node model of the STN/GPe circuit (inset) was fit to two different data sets: dataset *A* (blue) and dataset *B* (red) that were generated by different underlying models. Each estimation was performed 10 times with identical specification of prior distributions for all initializations. **(A)** Tracking of the goodness-of-fit (shown as -log_10_(-MSE_pooled_)) over the iterations demonstrated consistent convergence. Posterior estimates of the summary statistic were on average more accurate for dataset A than for B but were consistent across multi-starts **(B)** Optimization showed a consistent increase in the average precision (equivalent to a decrease in the logarithm of the inverse standard deviation of the data) of the posteriors indicating that data was informative in constraining parameter estimates. **(C)** Examination of the MAP estimates demonstrated a consistent inference of parameter values. Some parameters were drawn to common values with both data *A* and *B* (e.g. GPe time constant), whilst others show differences informed by the data (e.g. STN time constant). MAP values are given as log scaling parameters of the prior mean. The prior values were set to equal zero. Error bars give the standard deviations of the estimates across initializations. Asterisks indicate significant *t*-test for difference in means between parameters estimated from data *A* and *B*. **(D)** To visualize trajectories of the multi-starts, the high dimensional parameter space was reduced to two dimensions using multi-dimensional scaling (MDS). Evolutions of the means of the proposal parameters exhibit a clear divergence between data sets A and B that were significantly different (MANOVA, see main text). (For interpretation of the references to colour in this figure legend, the reader is referred to the web version of this article.)

This section of the results examines the face validity of parameter estimation of ABC i.e. that the scheme will (a) make a consistent estimation of posterior model parameters across multiple realizations of optimization; and (b) yield posterior estimates of parameters that are plausible given the known causes of the data. This is achieved using a multi-start procedure (Baritompa and Hendrix, 2005) described in the methods (Section 2.7.1) in which we initialize the algorithm eight times for two separate datasets generated by different underlying models. The results of the multi-starts are shown in Fig. 3.

The evolution of the objective function (MSE_pooled_) over the progress of the optimization is presented in Fig. 3A. Multi-starts of the optimization for both dataset A and B exhibited consistent degrees of error in the posterior summary statistics. In Fig. 3B, the average log-precision of the marginal densities is tracked over the progress of the optimization. These data show that across all initializations, the average precision of the posterior densities (1/σ = 20) was 2.5 times greater than those of the priors (1/σ = 8) demonstrating increased confidence in parameters estimates that were constrained by the data.

In Fig. 3C, we present the maximum a posterior (MAP) estimates for each parameter across the multi-starts. There are clear differences between parameters inverted upon the two separate sets of data (red versus blue bars; asterisks indicate significant t-tests). For instance, the mean GPe time constant (1st group of bars from the left) is smaller for data A compared to B. Other parameters were well-informed by the data, but not significantly different between either data sets (e.g. GPe sigmoidal slope; 2nd set of bars from the left). The accuracy of the parameter optimization procedure was assessed by comparing the MAP estimates to the known parameters of the underlying forward models. This can be seen in Fig. 3C where the parameter values are plot as crosses alongside the ABC posterior expectations. Estimates of STN and GPe time constants and the GPe → STN delay were well-recovered (the actual value falls within the spread of multi-start estimates). The slope of the nonlinear activation function however was poorly recovered showing consistent over-estimation, likely due to the real value falling far out of the bounds of the prior.

To estimate the internal-consistency of the parameter estimates, we applied a one-sample Hotelling test within an eight-fold, leave-one-out cross-validation to each of the MAP estimates from the multi-start. For both samples of parameters estimated from data A and B we find there to be a 0% rejection of the null hypothesis that the mean of the fold is significantly different from that of the left-out sample. This result indicates that every initialization of the multi-start fell within the variance defined by the remainder of the multi-starts. This does not test whether the variance is unacceptably large, we test this in part by examining (ii): that the posteriors were differentiated by the data on which they were estimated, using a Szekely and Rizzo energy test. We find there to be a significant difference in the means of the two samples (Φ = 5.13; *P* = 0.001). This finding is supported by a MANOVA test that demonstrates that the two data sets are significantly segregated by their posterior parameter means (*D* = 1, *P* < 0.001). This suggests that the spread of estimates was, at least, sufficient to distinguish posteriors derived from different underlying generative models. Visualization of the parameter space using multidimensional scaling (MDS; Fig. 3D) confirms the segregation of the posterior samples into two clusters determined by the datasets from which they are estimated. These results confirm that the ABC optimized posteriors are consistent across multiple initializations and that the output is determined by differences in the underlying model generating the given data.

### 3.3 Testing face validity of the model comparison approach

**Fig. 4:**
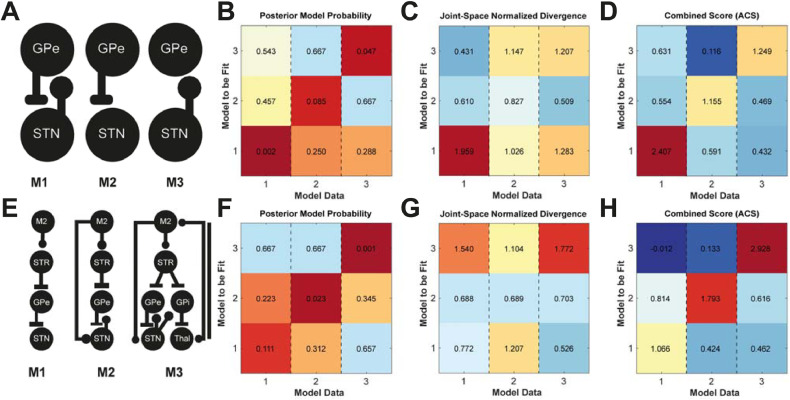
**Testing face validity of the ABC model comparison approach to model identification.** Confusion matrices were constructed by fitting the three models of the STN/GPe circuit. Synthetic data was generated using the fitted models and then the three original models were fitted back to the synthetic data to test whether model comparison could identify the generating model. **(A)** Schematic of neural mass model to be fitted. Annotations of connections indicate the presence of each for models 1–3. **(B)** Matrix of posterior model probabilities 1-*P(M|D)* computed normalized across the joint model space (for each column demarcated by dashed lines). **(C)** Matrix of normalized divergences of posteriors from priors (see second term of Eq. (9)). **(D)** Combined scoring to simultaneously account for model accuracies and divergence (ACS). Large values indicate better fits with more parsimonious posteriors (small D_KL_). **(E-F)** Same as for (A-D) but for the more complex model set.

To verify that the face validity of the model comparison approach i.e. that it can identify the correct structure of the generative model of the data we constructed a confusion matrix (as detailed in the methods Section 2.7.2), first using variations on the STN/GPe model presented in the previous sections and shown in Fig. 4A. In the case of correct model identification, the best model scores should lay along the diagonal of the confusion matrix.

In Fig. 4B we present the posterior model probabilities *P(M|D)* (see methods for details of its calculation). When normalizing across the joint space to compute the marginal posterior probability of a model, we consider only the three models tested per dataset (i.e. the sum of the probabilities across each column of Fig. 4B, C, F and G are equal to one). This analysis demonstrates that, in terms of accuracy, the most probable models lie on the diagonal of the confusion matrix showing that the posterior accuracies are sufficient to correctly identify the generating model. In Fig. 4C, we present the model complexity in terms of a proportion of the sum divergence across all models (i.e. the second term of Eq. (9)). These analyses show that the divergence of each model's posteriors from priors (so called *complexity,* measured in terms of the D_KL_). In the case of model 1 (which is the most flexible in terms of numbers of free parameters) there are inflated divergences in the first column that result from a large deviation of posteriors when attempting to fit the data generated from the alternative models. This shows that a post-hoc analysis of divergence of the posterior (using D_KL_) can be used to discriminate models which have been overfitted. When combining these two measures into the ACS metric (summarising model accuracy minus complexity) in Fig. 4D, it is seen that the best fitting models are still correctly identified even when accounting for the increased complexity of posterior parameter densities. Note that the most flexible model (model 1) was unable to fit data from models 2 and 3, this occurred as the required posteriors to achieve effectively decouple STN/GPe feedback were very far from the model 1 priors on connectivity. This is a known limitation of ABC, please see the discussion.

To determine whether this face validation held for larger models, we performed an identical analysis with three models ranging in complexity (Fig. 4E). Model accuracy was again highest along the diagonal (Fig. 4F), with the complexity adjusted goodness-of-fit (ACS; Fig. 4H) maintaining correct model identification. These results demonstrate that the model comparison approach can properly identify models from which the data originated, thus providing a face validation of the model comparison procedures.

### 3.4 Scaling up to larger model spaces: application to models of the cortico-basal ganglia-thalamic circuit

**Fig. 5:**
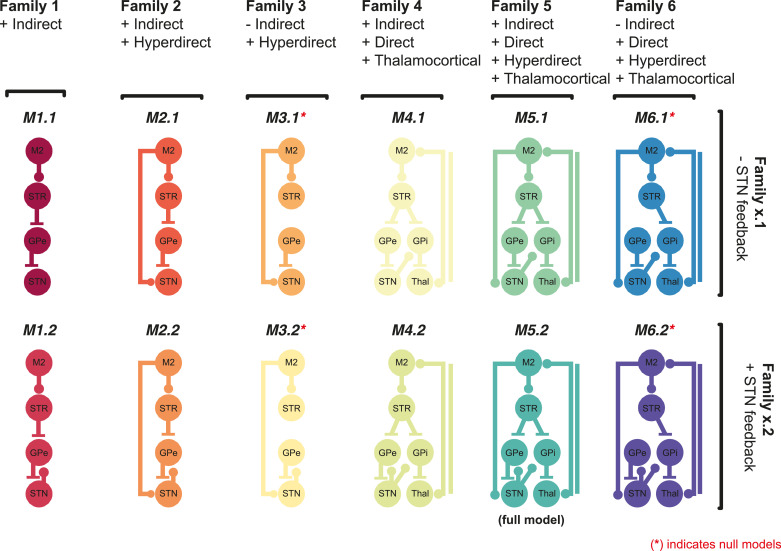
**Illustration of the model space of the cortico-basal-ganglia network fitted with ABC and compared with Bayesian model selection.** The model space comprises six families which can be further subdivided into two subfamilies yielding 12 models in total. Family (1) models the indirect pathway; family (2) contains models with both the indirect and hyperdirect pathways; family (3) contains models with the hyperdirect pathway but not indirect pathway; family (4) contains models with the indirect, direct and thalamocortical pathways; family (5) contains models with indirect, direct hyperdirect, and thalamocortical pathways; family (6) contains models with hyperdirect, direct and thalamocortical pathway but no indirect pathway. Finally, each family comprises two sub-families that either exclude (Mx.1) or include (Mx.2) subthalamopallidal feedback excitation. Excitatory projections are indicated by ball-ended connections, whilst inhibitory connections are flat-ended. (For interpretation of the references to colour in this figure legend, the reader is referred to the web version of this article.)

Finally, we applied the ABC framework to a larger and more complex model space to test the scalability of the methodology. Specifically, we devised a set of 12 models (illustrated in Fig. 5) incorporating combinations of pathways in the cortico-basal ganglia-thalamic circuit amongst a set of six neural populations motor cortex (M2); striatum (STR), GPe, STN, and thalamus (Thal.). Models were split into sets including/excluding the indirect (M2 → STR → GPe → STN); hyperdirect (M2 → STN); and thalamocortical relay (M2↔Thal.). Models were further subdivided to include or exclude the subthalamo-pallidal feedback connection (STN → GPe; models prefixed M *x*.2 to denote inclusion of the connection). For a full description and defence of the model choices please see methods Section 2.7.3. These models were fit individually to the empirical data and then model comparison used to determine the best candidate model.

**Fig. 6:**
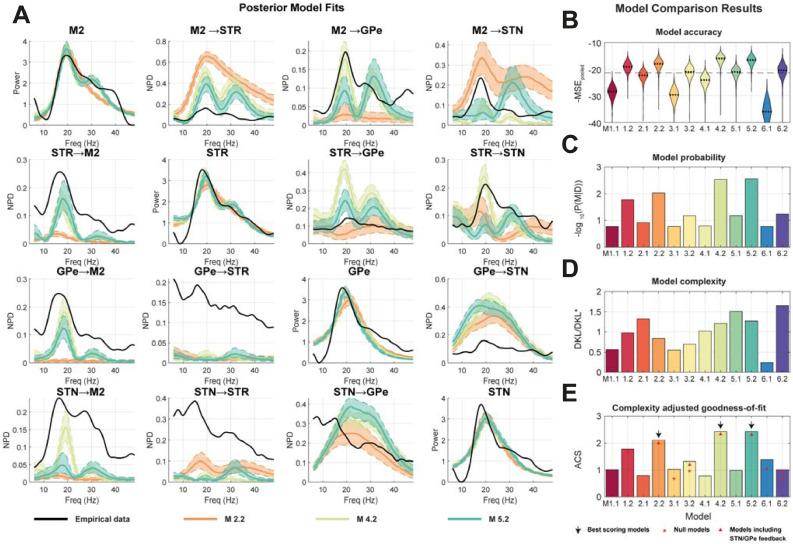
**Scaling up the ABC model comparison framework – investigating models of the cortico-basal ganglia-thalamic network.** 12 competing models (six families subdivided each into two sub-families) were fitted to empirical data from Parkinsonian rats. Models were fitted to summary statistics of recordings from the motor cortex (M2), striatum (STR), subthalamic nucleus (STN), and external segment of the globus pallidus (GPe). Models were first fit using ABC to estimate the approximate posterior distributions over parameters. To assess relative model performances, 1000 draws were made from each model posterior and corresponding data was simulated. **(A)** The posterior model fits for the top three performing models are shown, with autospectra on the diagonal and NPD on the off-diagonal (M 5.2 in light green; M 4.2 in turquois; and 1.2 in red). Bounds indicate the interquartile range of the simulated features. **(B)** Violin plots of the distributions of model accuracies (MSE_pooled_) of the simulated pseudo-data from the empirical data. **(C)** The acceptance probability approximation to the model evidence 1-*P(M|D)* is determined by computing the number of samples from the posterior that exceed the median model accuracy (MSE_pooled_). **(D)** The joint space normalized Kullback-Leibler divergence of the posterior from prior is shown for each model (for formulation see second term of Eq. (9)). Large values indicate high divergence and overfitting. **(E)** Combined scores for accuracy and divergence from priors using ACS. (For interpretation of the references to colour in this figure legend, the reader is referred to the web version of this article.)

In Fig. 6 we show the resulting model fits and then the subsequent model comparison done in order to determine the best model or set of models from the proposed model space. From visual inspection of the fits to the data features in Fig. 6A as well as the distribution of posterior model accuracies in Fig. 6B there is a wide range of model performances with regards to accurate fitting of the models to the data. Inspection of the posterior model fits to the data features in 6A shows the best fitting model (M 5.2) was able to account for the multiple peaks in the autospectra at around 20 Hz and 30 Hz. There was however a systematic underestimation of the directed functional connectivity (NPD) from subcortex to cortex. When the MSE_pooled_ of the fitted models was segregated between their autospectra and functional connectivity, we found that spectra were more accurately fit than for the power.

In all cases the models containing the subthalamopallidal excitatory connection (M *x*.2) performed better than those without (M *x*.1) and in good agreement with the known Parkinsonian electrophysiology (Cruz et al., 2011). Notably we found that the model families (3) and (6)- the null models- yielded poor accuracy with many of the posterior distributions of model MSE_pooled_ falling far below the median of the whole model space (Fig. 6B) that translates to a reduced model probability (Fig. 6C). In the case of M 6.2 we see that this is accompanied by a high KL divergence (Fig. 6D). M 4.2 and 5.2 are the strongest models, with distributions of fits tightly clustered around high values yielding high model evidences. This suggests the importance of including thalamocortical feedback connections in the model. Scoring with ACS suggests model 4.2 is the best of the models due to the smaller model complexity (parameter divergence from prior). These results further underwrite our face validation of the ABC procedure by demonstrating that its posterior estimates of model probability match well with the known neurobiology of the circuit.

## 4 Discussion

### 4.1 Summary of the results

In this paper we formulated a novel framework (Fig. 1) for the inverse modelling of neural dynamics based upon the ABC-SMC algorithm (Toni et al., 2009). We have provided a face validation of this method when applied to models and data types typically encountered in systems neuroscience. We first demonstrated that the algorithm converges to yield best fit approximations to the summary statistics of empirical data to yield posterior estimates over parameters (Fig. 2). We assessed the accuracy of parameter estimation by confirming that posterior estimates were plausible given the parameters that were known to generate the data (Fig. 3). Additionally, we used a multi-start procedure to demonstrate that the optimization was robust to local minima and thus generalizable across different realizations of the data. Next, we examined the face validity of the model selection procedures (Fig. 4). These results demonstrated that the model comparison approach can reliably identify the model that generated the data, even in cases in which more complex models (going up to 6 sources) were included. Finally, we demonstrated the capacity for the framework to investigate the structure of real-world neuronal circuits using a set of models of the cortico-basal-ganglia-thalamic circuit fit to empirical data (Fig. 5). Conclusions drawn from this model comparison matched well with the known neurobiology and further underwrite the feasibility of applying this method to answer biologically relevant problems (Fig. 6).

### 4.2 ABC for parameter estimation of neural circuit models

ABC has established itself as a key tool for parameter estimation in systems biology (Excoffier, 2009; Ratmann et al., 2009; Toni et al., 2009; Turner and Sederberg, 2012; Liepe et al., 2014) but is yet to see wide adoption in systems neuroscience. It is known that ABC will not perform well under certain conditions (for a review see Sunnåker et al., 2013). Specifically, it has been shown that the simplest form of the ABC algorithm, based upon an rejection-sampling approach, is inefficient in the case where the prior densities lie far from the true posterior (Lintusaari et al., 2016). This problem is alleviated to some degree in biological models where a good amount of a priori knowledge regarding plausible model structures or parameter values exists. This motivates the use of neurobiologically grounded models over phenomenological models where often the ranges of potential parameter values are unknown.

A caveat of simulation-based inverse modelling concerns the timescale of the simulation and the data features to be fitted. Necessary finite time observations can preclude the examination of slow modes or switching behaviour^1^ occurring at a time-scale beyond that captured in the empirical recording or simulation duration (see also discussion below regarding sufficiency of the summary statistics). In the deployment of ABC here, we use a model cast in a set of stochastic delay differential equations, in which the finite time realization of each noise process will lead to differences in the trajectory of the system between instances (i.e. *forward uncertainty*). In these cases, optimization with ABC will be drawn towards regions of parameter space where stochasticity does not result in large deviations between realizations as this will result in increased uncertainty in the posterior parameter estimates. Corroborating this, it was found that independent realizations of the stochastic model led to highly consistent summary statistics (appendix III).

Furthermore, the consistency in parameter estimates between multi-starts can also be taken as evidence of low *inverse uncertainty* as the values of estimated parameter did not deviate significantly between realizations of the underlying generative process. It would be of interest (but beyond of the scope of this paper) to evaluate the extent to which the variance between experimental samples (e.g. recordings from different animals within the same experimental treatment, or changes in sensor noise) can affect the consistency of parameter estimates (i.e. an examination of predictive validity). Schemes exist for DCM where data features may be weighted in terms of the estimated noise term, a similar extension is likely to be of use for ABC inverted models, especially in the case where multiple types of summary statistics are combined.

### 4.3 Sufficiency of the summary statistic and ABC model selection

The selection of summary statistics are known to be a vital factor in determining the outcomes of ABC estimated posterior (Beaumont et al., 2002; Sunnåker et al., 2013), as well as in model selection that where insufficiency of the statistic can affect models non-uniformly (Robert et al., 2011). Thus we can only interpret the results of a model comparison in terms of each model's capacity to explain the given summary statistic as an abstraction to the complete data. The choice of summary statistic will always introduce a degree of parameter non-identifiability, for instance an investigation of model behaviours involving switching or chaotic dynamics are unlikely to accurately identify the responsible parameters given a feature such as Fourier spectrum. In this work we used the directed functional connectivity (NPD) as a data feature by which to constrain our model(s) rather than the complex cross spectra (from which it can be derived). The NPD exhibits robustness to zero-lag effects arising from volume conduction that in turn simplifies the estimation of mixing terms in the observer model. In our validation here, we showed that the feature was sufficient to recover known parameters, but likely entails an increase in the degree of non-identifiability that could be examined in future work.

Furthermore, sampling approaches to the estimation of marginal likelihoods in order to perform Bayesian model comparison are challenging to compute (Chib, 1995) and common approximations have been demonstrated to be poor (Penny, 2012). Furthermore, sampling approximations to the model evidence such as that described here are highly dependant upon the distance from the true posterior and the sufficiency of the summary statistic. Further work would need to be done in order to understand how the ABC estimates of model evidence are limited by non-vanishing error tolerance (i.e. ϵ≠0) in which posteriors are only approximate (Dean et al., 2014).

### 4.4 Future directions for ABC and mesoscale neural modelling

This work follows on from a number of previous works that have performed inference from large scale models of brain activity and spectral domain summary statistics of neural recordings such as their cross-spectra or functional connectivity (Valdes et al., 1999; Rowe et al., 2004; van Albada et al., 2010; Friston et al., 2012; Hadida et al., 2018; Hashemi et al., 2018). Whilst similar in their aims, the computational challenge of the inverse problem has meant that the techniques adopted to solve it have dictated the types of questions to which they can be applied. Previous approaches to constraining models from spectral features have often bypassed finding explicit numerical solutions to models, instead opting to approximate dynamics by estimating the system's transfer function around a local-linearization (Valdes et al., 1999; Rowe et al., 2004). Beyond reducing the computational burden of numerical integration, this approach also facilitates the use of techniques such as variational Bayes (Friston et al., 2012) by ensuring that posterior densities conform to a multivariate Gaussian (the Laplace assumption).

Whilst this technique has proven powerful (e.g. Moran et al., 2011; Bastos et al., 2015), it precludes the examination of highly nonlinear models that exhibit structural instabilities (i.e. bifurcations or phase transitions) that will result in a non-convex cost function, and are thus unlikely to conform to the Laplace assumption. Importantly these bifurcations are known to exist in the neural mass models of the type used here (Aburn et al., 2012) and have been demonstrated to yield multimodal posteriors (Hadida et al., 2018). It would be of future interest to systematically delineate the conditions for when the above approximations. For instance, a comparison of posterior parameter estimates computed between ABC and DCM (i.e. a construct validation), in a model approaching a transition point would address the question of what approach is best suited to a particular modelling scenario.

Current approaches to the inverse modelling phenomena such as state transitions or time dependant fluctuations with DCM discretize these phenomena into either sliding windows (Rosch et al., 2018) or a succession states that evolve according to a matrix of transition probabilities (Zarghami and Friston, 2020). This follows from an assumption that time varying behaviour can be separated into fast local dynamics which are then under the control of some slow mode that dictates the succession (Rabinovich et al., 2012). Whilst this approach is useful for understanding the states, it somewhat abstracts the mechanisms that lay behind the transitions, whether that be due to slow changes in connectivity or parameters (e.g. plasticity), evolution of a slow variable (c.f. an order-parameter; Haken et al., 1985), or switching induced by stochastic drives to a model. Examination of these transitions are, for instance, important in models looking to interact with ongoing brain states through stimulation (see for instance West et al., 2020a). The framework described here provides an opportunity to investigate the mechanism behind these transitions and paves the way for future studies investigating the types of mechanisms that underwrite the statistics of for instance electrophysiological bursts (Powanwe and Longtin, 2019; Duchet et al., 2020) or neural microstates (Baker et al., 2014). Previous work has shown that ABC is well suited to applications using highly nonlinear or stochastic systems (see Toni et al., 2009 for an example).

## Conclusions

Overall, we have introduced a framework for parameter estimation and model comparison that draws upon a number of recent developments in simulation-based inference that make it attractive to the inverse modelling of large-scale neural activity. This framework provides a robust method by which large scale brain activity can be understood in terms of the underlying structure of the circuits that generate it. This scheme avoids making appeals to local-linear behaviour and thus opens the way to future studies exploring the mechanisms underlying itinerant or stochastic neural dynamics. We have demonstrated that this framework provides consistent estimation of parameters over multiple instances; can reliably identify the most plausible model that has generated an observed set of data; and given an example application demonstrating the potential for this framework to answer neurobiologically relevant questions. Whilst this paper constitutes a first validation and description of the method, more work will be required to establish its validity in the context of more complex models as well as statistics of time-dependant properties of neural dynamics.

## Data availability statement

All scripts for the analyses presented here can be found in a GitHub repository (https://github.com/twestWTCN/ABCNeuralModellingToolbox; release v1.0). A full list of script dependencies, toolboxes used, their authors, and links to their original source code used can be found in the supplementary data. The group level experimental data used in this paper is available upon reasonable request.

## CRediT authorship contribution statement

**Timothy O. West:** Conceptualization, Methodology, Software, Validation, Investigation, Writing – original draft, Writing – review & editing, Visualization. **Luc Berthouze:** Methodology, Resources, Writing – review & editing, Supervision, Funding acquisition. **Simon F. Farmer:** Conceptualization, Writing – review & editing, Supervision, Funding acquisition. **Hayriye Cagnan:** Resources, Writing – review & editing, Supervision, Funding acquisition. **Vladimir Litvak:** Conceptualization, Methodology, Resources, Writing – review & editing, Supervision, Funding acquisition.

## Appendix Supplementary materials

Image, application 1

## Footnotes

1 In appendix III we investigated whether the posterior models exhibited any of these dynamics for which we found no evidence.
