# Supplementary material for: Inference of brain networks with approximate Bayesian computation – assessing face validity with an example application in Parkinsonism
Source: Neuroimage. 2021 Aug 1;236:118020. doi: 10.1016/j.neuroimage.2021.118020 (PMC8270890; doi:10.1016/j.neuroimage.2021.118020)
Supplement: Supplementary file 1 [file mmc1.docx]

# Supplementary Information

# Simulation of Neural Model

### Notation

We denote spike rates by *x*, membrane potentials by *v*, synaptic gains by *H*, time constants by $\tau$, and external inputs by *A*. First order derivatives are indicated using Newton’s notation. Subscripts for intrinsic parameters/states denote the *i*^th^ population. The total output activity for each k^th^ _­_source, is given by $V_{k}$. Throughout we will use a plain letter for a vector (e.g. y) and a letter with a subscript for a vector element (e.g. y_i_).

### Modelling of Neural Dynamics

In order to simulate neural signals generated by a neuronal source, we used a coupled network of neural mass models that simulate field potentials generated by synchronized activity of large ensembles of homogenous neurons (Lopes da Silva, 1991; Jansen and Rit, 1995). These models rest on the assumption that an incoming volley of spikes arriving at a population can be converted to a post synaptic potential by convolution with a synaptic response kernel. Following temporal integration of the post synaptic potentials, the population can then in turn generate a spike density with a sigmoidal mapping of membrane voltage to spike output frequency. Each of the *i^th^* populations may be coupled via *intrinsic* connectivity to simulate the dynamics within cortical columns. These intrinsically coupled populations are divided into *k* sources that are themselves *extrinsically* connected to simulate inter-areal coupling (Wendling et al., 2000; David and Friston, 2003). The *i^th^* population within the *l^th^* source is given generally by the form:

$\dot{v_{i}}=x_{i}$,

$$\dot{\boldsymbol{x}_{\boldsymbol{i}}}\boldsymbol{=}\frac{\boldsymbol{H}_{\boldsymbol{i}}}{\boldsymbol{\tau}_{\boldsymbol{i}}}\left( \boldsymbol{u}_{\boldsymbol{i}}\boldsymbol{+}\boldsymbol{S}_{\boldsymbol{i}}\left( \boldsymbol{A}_{\boldsymbol{l}} \right) \right)\boldsymbol{-}\frac{\boldsymbol{2}}{\boldsymbol{\tau}_{\boldsymbol{i}}}\boldsymbol{x}_{\boldsymbol{i}}\boldsymbol{-}\frac{\boldsymbol{1}}{\boldsymbol{\tau}_{\boldsymbol{i}}^{\boldsymbol{2}}}\boldsymbol{v}_{\boldsymbol{i}}\boldsymbol{,}$$

Equation s1

where the average postsynaptic membrane potential of the $i$th population is given by $v_{i}$, and is parameterized by a synaptic gain $H_{i}$ and a lumped post synaptic time constant $\tau_{i}$. The input to the mass is given between the outer set of brackets and comprises some background noise $u_{i}$ plus a combined input from the other *K* sources. Populations within each *k^th^* source (e.g. one cortical column) are intrinsically coupled (instantaneously) via gain factors *H* that allow inhibitory and excitatory populations to interact. Inhibitory cells have negative connection weights and vice versa for excitatory cells.

To couple distant sources (extrinsic connectivity), the total input to the *l^th^* source is the weighted sum (set by the adjacency matrix $\omega)$ of inputs across all *K* populations:

$$A_{l}=\sum_{l=1}^{K} \omega_{l,k}V_{k}$$

Equation s2

where $V_{k}$ is the total output from the *k^th^* source (see below). Membrane potentials are converted to spike densities via the sigmoid operator:

$$S_{i}(A_{l})=1/(1+e^{-R_{i}A_{l}}),$$

Equation s3

which is parameterised by $R_{i}$ to determine the slope of the activation function (a parameter specific to the *i^th^* population) and effectively models the variance of the population’s firing thresholds. The final terms of equation s1 relate to the fact that these equations are equivalent to the convolution operation of an exponential kernel (see Jansen and Rit 1995 for details of the derivation).

To create the full model describing the basal ganglia and motor cortex, masses are coupled with a structure outlined in the schematic in the full model shown in figure 6. We model inhibitory connections by flipping the sign on the adjacency matrix, such that they have a subtractive influence. Connectivity was simulated between sources (extrinsic connectivity), the neural activity (given by *V*) propagates from the *k^th^* to the *l^th^* source according to a weighted adjacency matrix with entries $\omega_{l,k}$ indicating the strength and polairty of the connection. The adjacency matrix of the full model is given below

$$\omega=\left[ \begin{matrix} 0 & 0 & 0 & 0 & 0 & \omega_{1,6} \\ \omega_{2,1} & 0 & 0 & 0 & 0 & 0 \\ 0 & \omega_{3,2} & 0 & \omega_{3,4} & 0 & 0 \\ \omega_{4,1} & 0 & \omega_{4,3} & 0 & 0 & 0 \\ 0 & \omega_{5,2} & 0 & \omega_{5,4} & 0 & 0 \\ 0 & 0 & 0 & 0 & \omega_{6,5} & 0 \end{matrix} \right]$$

Equation s4

where column 1 gives connections projecting from M2; column 2 from the STR; column 3 from the GPe; column 4 from the STN; column 5 from the GPi; and column 6 from the thalamus. Equivalently the rows give the weights of the input to the populations. Variants of this full model can then be created by adjusting the parameters or removing coefficients $\omega_{ji}$ from the matrix.

## Transmission Delays

We incorporate finite transmission delays by formulating the state space equations to explicitly depend on the past activity of the source from which the connection originated. This was achieved by modifying the extrinsic connectivity matrices by indexing past values with a matrix $D$ with elements $D_{k,l}$ specifying the delay for connection of source *k* to *l*. Thus, the total external input to source $l$ at time $t$ is:

$$A_{l}\left( t \right)=\sum_{k=1}^{K} \omega_{l,k}V_{k}\left( t-D_{l,k} \right),$$

Equation s5

with the constraint that $D_{l,k}>0$.

## State Equations of Full Model

In total there are 14 state equations that are an adaption of the full model described in van Wijk et al. (2018). Each mass (population) comprises two equations (decomposing the second order differential equations into two first order ones) that describes the voltage change of the population. Again, the model is divided into *K* extrinsically coupled sources (M2, STR, GPe, STN, GPi, and Thal.) that each comprise a set of intrinsically coupled populations.

The 1^st^ source (M2) model consists of 4 populations of neurons (8 states). Each layer is connected via intrinsic connectivity with synaptic gain parameters (*H*). All populations in the motor cortex have a self-inhibiting connection. In order to notate intrinsic connections from the *j^th^* to the *i^th^* population, we extend our subscripts for synaptic gains to *H_i,j_*. All layers receive independent stochastic inputs *u_i_*. The cortical source comprises:

1. A middle layer composed of middle pyramidal cells with inhibitory self-connection (with strength parameterized by H_1,1_):

$\dot{v_{1}}=x_{1}$,

$\dot{x_{1}}=\frac{1}{\tau_{1}}\left( -H_{1,1} S_{1}\left( v_{1} \right)-H_{1,3} S_{1}\left( v_{3} \right)+H_{1,2} S_{1}\left( v_{2} \right)+S_{1}(A_{1})+u_{1} \right)-\frac{2}{\tau_{1}}x_{1}-\frac{1}{\tau_{1}^{2}}v_{1}$;

Equation s9

1. A supra-granular layer composed of superficial pyramidal cells with inhibitory self-connection (with strength parameterized by H_2,2_):

$\dot{v_{2}}=x_{2}$,

$\dot{x_{2}}=\frac{1}{\tau_{2}}\left( {-H}_{2,2} S_{2}\left( v_{2} \right)+H_{2,1} S_{2}\left( v_{1} \right)-H_{2,3} S_{2}\left( v_{3} \right)+H_{2,4} S_{2}\left( v_{4} \right)+u_{2} \right)-\frac{2}{\tau_{2}}x_{2}-\frac{1}{\tau_{2}^{2}}v_{2}$;

Equation s10

1. The supra-granular layer also contains a separate inhibitory interneuron population, again with an inhibitory self-connection (with strength parameterized by H_3,3_):

$\dot{v_{3}}=x_{3}$,

$\dot{x_{3}}=\frac{1}{\tau_{3}}\left( {-H}_{3,3} S_{3}\left( v_{3} \right)+H_{3,1} S_{3}\left( v_{1} \right)+H_{3,4} S_{3}\left( v_{4} \right)+H_{3,2} S_{3}\left( v_{2} \right)+u_{3} \right)-\frac{2}{\tau_{3}}x_{3}-\frac{1}{\tau_{3}^{2}}v_{3}$;

Equation s11

1. Finally, the infra-granular layer is made up of deep pyramidal cells also with an inhibitory self-connection (with strength parameterized by H_4,4_)

$\dot{v_{4}}=x_{4}$,

$\dot{x_{4}}=\frac{1}{\tau_{4}}\left( {-H}_{4,4} S_{4}\left( v_{4} \right)-H_{4,3} S_{4}\left( v_{3} \right)+H_{4,2} S_{4}\left( v_{2} \right)+u_{4} \right)-\frac{2}{\tau_{4}}x_{4}-\frac{1}{\tau_{4}^{2}}v_{4}$.

Equation s12

Overall, the output of the cortex is equal to the voltage in the deep pyramidal layer, thus:

$V_{1}=v_{4}$.

Equation s13

The 2^nd^ source (STR), is modelled as a single inhibitory population and self-inhibitory connection (with strength parameterized by H_5,5_):

$\dot{v_{5}}=x_{5}$,

$\dot{x_{5}}=\frac{1}{\tau_{5}}\left( {-H}_{5,5} S_{5}\left( v_{5} \right)+S_{5}(A_{2})+u_{5} \right)-\frac{2}{\tau_{5}}x_{5}-\frac{1}{\tau_{5}^{2}}v_{5}$;

Equation s14

and total output:

$V_{2}=v_{5}$.

Equation s15

The 3^rd^ source (GPe) is modelled as a single inhibitory population:

$\dot{v_{6}}=x_{6}$,

$\dot{x_{6}}=\frac{1}{\tau_{6}}\left( S_{6}\left( A_{3} \right)+u_{6} \right)-\frac{2}{\tau_{6}}x_{6}-\frac{1}{\tau_{6}^{2}}v_{6}$,

Equation s16

and total output:

$V_{3}=v_{6}$.

Equation s17

The 4^th^ source (STN) is modelled as a single excitatory population:

$\dot{v_{7}}=x_{7}$,

$\dot{x_{7}}=\frac{1}{\tau_{7}}\left( S_{7}(A_{4})+u_{7} \right)-\frac{2}{\tau_{7}}x_{7}-\frac{1}{\tau_{7}^{2}}v_{7}$,

Equation s18

and total output:

$V_{4}=v_{7}$.

Equation s19

The 5^th^ source (GPi) is taken to be a single inhibitory population:

$\dot{v_{8}}=x_{8}$,

$\dot{x_{8}}=\frac{1}{\tau_{8}}\left( S_{8}\left( A_{5} \right)+u_{8} \right)-\frac{2}{\tau_{8}}x_{8}-\frac{1}{\tau_{8}^{2}}v_{8}$,

Equation s20

and total output:

$V_{5}=v_{8}$.

Equation s21

The 6^th^ source (Thal.) is taken to be a single excitatory population with self-inhibition (with strength parameterized by H_9,9_):

$\dot{v_{9}}=x_{9}$,

$\dot{x_{9}}=\frac{1}{\tau_{9}}\left( -H_{9,9}S_{9}\left( v_{9} \right)+S_{9}\left( A_{6} \right)+u_{9} \right)-\frac{2}{\tau_{9}}x_{9}-\frac{1}{\tau_{9}^{2}}v_{9}$,

Equation s22

and total output:

$V_{6}=v_{9}$.

Equation s23

## Integration of Stochastic Delay Differential Equations

The model also incorporates a stochastic input $u_{i}$for each population which represents endogenous background activity. This input is given by:

$$u_{i}=C_{i}W,$$

Equation s6

where

$$W\sim N\left( 0,\sigma\right),$$

Equation s7

and $C_{i}$ represents a gain factor on the noise scaling the noise for population $i$. The noise $W$is drawn from a zero-mean normal distribution, with a standard deviation $\sigma$ that is set for the whole model. In the model presented here the stochastic innovations are independent of the state variable (i.e. they are additive) and a Euler-Maruyama (EM) scheme with a suitably small step size (*h* = 0.001ms*;* less than half of the fastest time constant) is appropriate. See appendix II for an examination of the choice of stepsize This numerical scheme has been demonstrated to yield accurate results in similar models (Ableidinger et al., 2017; Palmigiano et al., 2017). A formal assessment of the convergence of the EM scheme is beyond the remit of this paper but we refer the technical reader to (Baker and Buckwar, 2000; Buckwar, 2000). For additive noise, this scheme follows on naturally from forward Euler and deploys a rescaling of the stochastic component by the square-root of the integration step $h$ to ensure fluctuations are obey a proper Weiner process(as per Hansen et al. 2006):

$\hat{u_{i}}=u_{i}\surd h$.

Equation s8

To allow for settling of state equations, we set the initial states to be equal to zero, and then remove the initial transient (3s) as a burn-in.

# Formulation of Objective Function

The objective function computes the error between the summary statistics of the simulated pseudo-data and empirical data ($\rho\left( \mu_{n},\mu_{0} \right)$). To do this we use the pooled mean squared error:

$$MSE_{pooled}=-\frac{1}{W}\sum_{w=1}^{W} \left( \frac{1}{X^{w}}\sum_{x=1}^{X^{w}} \left( \mu_{n}^{w}(x)-\mu_{0}^{w}(x) \right) \right)$$

Equation s8

where $X^{w}$ is the length of the *w^th^* data feature, over *W* features. For instance, in the case of the data features used in this paper, *W = 4* for two signals, as there are two power spectra and two directed functional connectivity spectra.

# Table of Prior Parameter Values

| Parameter | Mean Value(s) | Units | Log Precision^[[1]](#footnote-1)^ |
| --- | --- | --- | --- |
| Cortical Model |  |  |  |
| Intrinsic Connectivity of middle layer:$\boldsymbol{H}_{\boldsymbol{1,i}}$ | [400 400 400 0] | s^-1^ | 1/4 σ^2^ |
| Intrinsic Connectivity of supra-granular (pyramidal) layer: $\boldsymbol{H}_{\boldsymbol{2,i}}$ | [800 400 400 400] | s^-1^ | 1/4 σ^2^ |
| Intrinsic Connectivity of supra-granular (inhibitory) layer: $\boldsymbol{H}_{\boldsymbol{3,i}}$ | [400 400 400 400] | s^-1^ | 1/4 σ^2^ |
| Intrinsic Connectivity of infra-granular layer: $\boldsymbol{H}_{\boldsymbol{4,i}}$ | [0 800 400 400] | s^-1^ | 1/4 σ^2^ |
| Time Constants:  $\boldsymbol{\tau}_{\boldsymbol{1\ldots4}}$ | [3 2 12 18] | ms | 1/4 σ^2^ |
| Sigmoid Slope: $\boldsymbol{R}_{\boldsymbol{1\ldots4}}$ | All 2/3 |  | 1/4 σ^2^ |
| STR Model |  |  |  |
| Self-Inhibition:$\boldsymbol{H}_{\boldsymbol{5,5}}$ | 400 | s^-1^ | 1/8 σ^2^ |
| Time Constant: $\boldsymbol{\tau}_{\boldsymbol{5}}$ | 8 | ms | 1/8 σ^2^ |
| Sigmoid Slope:$\boldsymbol{R}_{\boldsymbol{5}}$ | 2/3 |  | 1/4 σ^2^ |
| GPe Model |  |  |  |
| Self-Inhibition: | 0 | s^-1^ | 1/8 σ^2^ |
| Time Constant: $\boldsymbol{\tau}_{\boldsymbol{6}}$ | 8 | ms | 1/8 σ^2^ |
| Sigmoid Slope:$\boldsymbol{R}_{\boldsymbol{6}}$ | 2/3 |  | 1/8 σ^2^ |
| STN Model |  |  |  |
| Self- Inhibition: | 0 | s^-1^ | 1/8 σ^2^ |
| Time Constant: $\boldsymbol{\tau}_{\boldsymbol{7}}$ | 4 | ms | 1/8 σ^2^ |
| Sigmoid Slope:$\boldsymbol{R}_{\boldsymbol{7}}$ | 2/3 |  | 1/8 σ^2^ |
| GPi Model |  |  |  |
| Self- Inhibition: | 0 | s^-1^ | 1/8 σ^2^ |
| Time Constant: $\boldsymbol{\tau}_{\boldsymbol{8}}$ | 8 | ms | 1/8 σ^2^ |
| Sigmoid Slope:$\boldsymbol{R}_{\boldsymbol{8}}$ | 2/3 |  | 1/8 σ^2^ |
| Thal. Model |  |  |  |
| Self-Inhibition:  $\boldsymbol{H}_{\boldsymbol{9,9}}$ | 400 | s^-1^ | 1/8 σ^2^ |
| Time Constant: $\boldsymbol{\tau}_{\boldsymbol{9}}$ | 8 | ms | 1/8 σ^2^ |
| Sigmoid Slope:$\boldsymbol{R}_{\boldsymbol{9}}$ | 2/3 |  | 1/8 σ^2^ |
|  |  |  |  |
| Delays |  |  |  |
| M2 → STR: $\boldsymbol{D}_{\boldsymbol{5,4}}$ | 3 | ms | 1/4 σ^2^ |
| M2 → STN: $\boldsymbol{D}_{\boldsymbol{7,4}}$ | 3 | ms | 1/4 σ^2^ |
| STR → GPe: $\boldsymbol{D}_{\boldsymbol{6,5}}$ | 7 | ms | 1/4 σ^2^ |
| STR → GPi: $\boldsymbol{D}_{\boldsymbol{8,5}}$ | 12 | ms | 1/4 σ^2^ |
| GPe → STN: $\boldsymbol{D}_{\boldsymbol{7,6}}$ | 1 | ms | 1/4 σ^2^ |
| GPe → GPi: $\boldsymbol{D}_{\boldsymbol{8,6}}$ | 1 | ms | 1/4 σ^2^ |
| STN → GPe:$\boldsymbol{D}_{\boldsymbol{6,7}}$ | 3 | ms | 1/4 σ^2^ |
| STN → GPi: $\boldsymbol{D}_{\boldsymbol{8,7}}$ | 3 | ms | 1/4 σ^2^ |
| GPi → Thal.: $\boldsymbol{D}_{\boldsymbol{9,8}}$ | 3 | ms | 1/4 σ^2^ |
| Thal. → M2: $\boldsymbol{D}_{\boldsymbol{1,9}}$ | 3 | ms | 1/4 σ^2^ |
| M2 → Thal.: $\boldsymbol{D}_{\boldsymbol{9,4}}$ | 8 | ms | 1/4 σ^2^ |
|  |  |  |  |
| Connections |  |  |  |
| M2 → STR: $\boldsymbol{A}_{\boldsymbol{5,4}}$ | (+) 2000 | s^-1^ | 1/4 σ^2^ |
| M2 → STN: $\boldsymbol{A}_{\boldsymbol{7,4}}$ | (+) 2000 | s^-1^ | 1/4 σ^2^ |
| STR → GPe: $\boldsymbol{A}_{\boldsymbol{6,5}}$ | (-) 1600 | s^-1^ | 1/4 σ^2^ |
| STR → GPi: $\boldsymbol{A}_{\boldsymbol{8,5}}$ | (-) 1600 | s^-1^ | 1/4 σ^2^ |
| GPe → STN: $\boldsymbol{A}_{\boldsymbol{7,6}}$ | (-) 2000 | s^-1^ | 1/4 σ^2^ |
| GPe → GPi: $\boldsymbol{A}_{\boldsymbol{8,6}}$ | (-) 2000 | s^-1^ | 1/4 σ^2^ |
| STN → GPe:$\boldsymbol{A}_{\boldsymbol{6,7}}$ | (+) 2000 | s^-1^ | 1/4 σ^2^ |
| STN → GPi: $\boldsymbol{A}_{\boldsymbol{8,7}}$ | (+) 2000 | s^-1^ | 1/4 σ^2^ |
| GPi → Thal.: $\boldsymbol{A}_{\boldsymbol{9,8}}$ | (-) 1600 | s^-1^ | 1/4 σ^2^ |
| Thal. → M2: $\boldsymbol{A}_{\boldsymbol{1,9}}$ | (+) 1000 | s^-1^ | 1/4 σ^2^ |
| M2 → Thal.: $\boldsymbol{A}_{\boldsymbol{9,4}}$ | (+) 2000 | s^-1^ | 1/4 σ^2^ |
|  |  |  |  |
| Observation Model |  |  |  |
| Observation noise (per source) | [0.2 0.2 0.2 0.2] | scalar | 1 σ^2^ |

# Computation of Kullback-Leibler Divergence for Multivariate Normal Distribution

In order to compute the full multivariate divergence between posterior $P_{1}=P\left( \theta| D_{0} \right)=N(\mu_{1},\Sigma_{1})$ and prior distributions $P_{0}=P\left( \theta| D_{0} \right)= N(\mu_{0},\Sigma_{0})$ over parameters we make an approximation to a *k-*dimensional multivariate normal distribution by estimating the mean and covariance of *N* parameters drawn from the joint kernel estimate of the posterior. The Kullback-Leibler distance evaluated from the mean and covariance of the distributions:

$$KL\left( P_{1} | |P_{0} \right)=\frac{1}{2}\left( tr(\Sigma_{0}^{-1}\Sigma_{1})+\left( \mu_{0}-\mu_{1} \right)^{T}\Sigma_{1}^{-1}\left( \mu_{0}-\mu_{1} \right)-k+ln\left( \frac{det\Sigma_{0}}{det\Sigma_{1}} \right) \right)$$

Equation s24

# Computation of Pooled Mean Squared Error

To compute the distance between summary statistics computed from simulated data and that of the target data, we compute the pooled mean squared error:

$$MSE_{pooled}=\frac{1}{N_{f}}\sum_{n=1}^{N_{f}} \left( \frac{1}{N_{n}}\sum_{i=1}^{N_{n}} \left( \mu_{data}^{n,i}-y_{model}^{n,i} \right)^{2} \right)$$

where *N_f_* is the number of features (i.e. *N_f_* = 16 for data comprising 4 channels: 4 autospectra + 12 directed functional connectivity spectra), and *i* is the length of the data feature *n*.

Equation s24

# List of Toolboxes Used

We thank all authors of the toolboxes below:

| Toolbox Name | Author | License | Year | Accessed: |
| --- | --- | --- | --- | --- |
| allcomb | ‘Jos’ | BSD-3-Clause | 2018 | <https://uk.mathworks.com/matlabcentral/fileexchange/10064-allcomb-varargin> |
| boundedline-pkg | Kelly Kearney | MIT | 2015 | <https://github.com/kakearney/boundedline-pkg> |
| bplot | Jonathan C. Lansey | BSD-3-Clause | 2015 | <https://uk.mathworks.com/matlabcentral/fileexchange/42470-box-and-whiskers-plot-without-statistics-toolbox> |
| brewermap | Stephen Cobeldick | Apache 2.0 | 2014 | <https://github.com/DrosteEffect/BrewerMap> |
| export_fig | Oliver J. Woodford, Yair M. Altman | BSD-3-Clause | 2014 | <https://github.com/altmany/export_fig> |
| highdim | Brian Lau | GNU-3 | 2017 | <https://github.com/brian-lau/highdim> |
| hotellingT2 | Antonio Trujillo-Ortiz | BSD-3-Clause | 2002 | <https://uk.mathworks.com/matlabcentral/fileexchange/2844-hotellingt2> |
| linspecer | Jonathan C. Lansey | BSD-3-Clause | 2015 | <https://github.com/davidkun/linspecer> |
| neurospec 2.2 | David Halliday | GNU-2 | 2018 | <http://www.neurospec.org/> |
| ParforProgMon | Dylan Muir, Willem-Jan de Goeij, The MathWorks, Inc. | BSD-3-Clause | 2016 | <https://github.com/DylanMuir/ParforProgMon> |
| splitvec | Bruno Luong | BSD-3-Clause | 2009 | <https://uk.mathworks.com/matlabcentral/fileexchange/24255-splitvec> |
| SPM-12 | The FIL Methods Group | GNU-2 | 2020 | <https://www.fil.ion.ucl.ac.uk/spm/software/spm12/> |
| violin | Holger Hoffmann | BSD-3-Clause | 2015 | <https://uk.mathworks.com/matlabcentral/fileexchange/45134-violin-plot> |
| weightedcov | Liber Eleutherios | BSD-3-Clause | 2008 | <https://uk.mathworks.com/matlabcentral/fileexchange/37184-weighted-covariance-matrix> |

# Appendices

## Appendix I- Table of Methods Used

| Method | Notes | Reference(s) |
| --- | --- | --- |
| Convolution based neural mass models | Mean field approximation to homogenous neural population activity. | (Lopes da Silva et al., 1974; Jansen and Rit, 1995; David and Friston, 2003) |
| Neural mass model of the cortico-basal ganglia -thalamic circuit | Population model with structure and parameterization defended and explained in given references. | (Moran et al., 2011; van Wijk et al., 2018) |
| Likelihood free inference with Approximate Bayesian Computation | References are for introductions/tutorials | (Beaumont et al., 2002; Sunnåker et al., 2013; Sisson et al., 2018) |
| Sequential Approximate Bayesian Computation | Improvement on ABC to aid convergence and computational efficiency | (Beaumont et al., 2009; Toni et al., 2009) |
| Kernel density approximation to ABC marginals and copula estimation of dependence | We use a cross-validation log-likelihood optimization of the kernel density bandwidth for estimation of the marginals, with approximate maximum likelihood to fit a t-copula to estimate the joint. | (Li et al., 2017) |
| Model Selection with ABC Optimized Models | Using acceptance rates as approximation to marginal likelihood. | (Grelaud et al., 2009; Toni and Stumpf, 2009) |
| Electrophysiological recordings from Parkinsonian rats | Field Recordings made in experimental 6-OHDA model of Parkinsonism | (Mallet et al., 2008; Moran et al., 2011) |
| Non-parametric Directionality for directed functional connectivity estimates | Used as a summary statistic of between signal interactions. | (Halliday et al., 2016; West et al., 2020b) |

## Appendix II - Examination of Integration Step-size


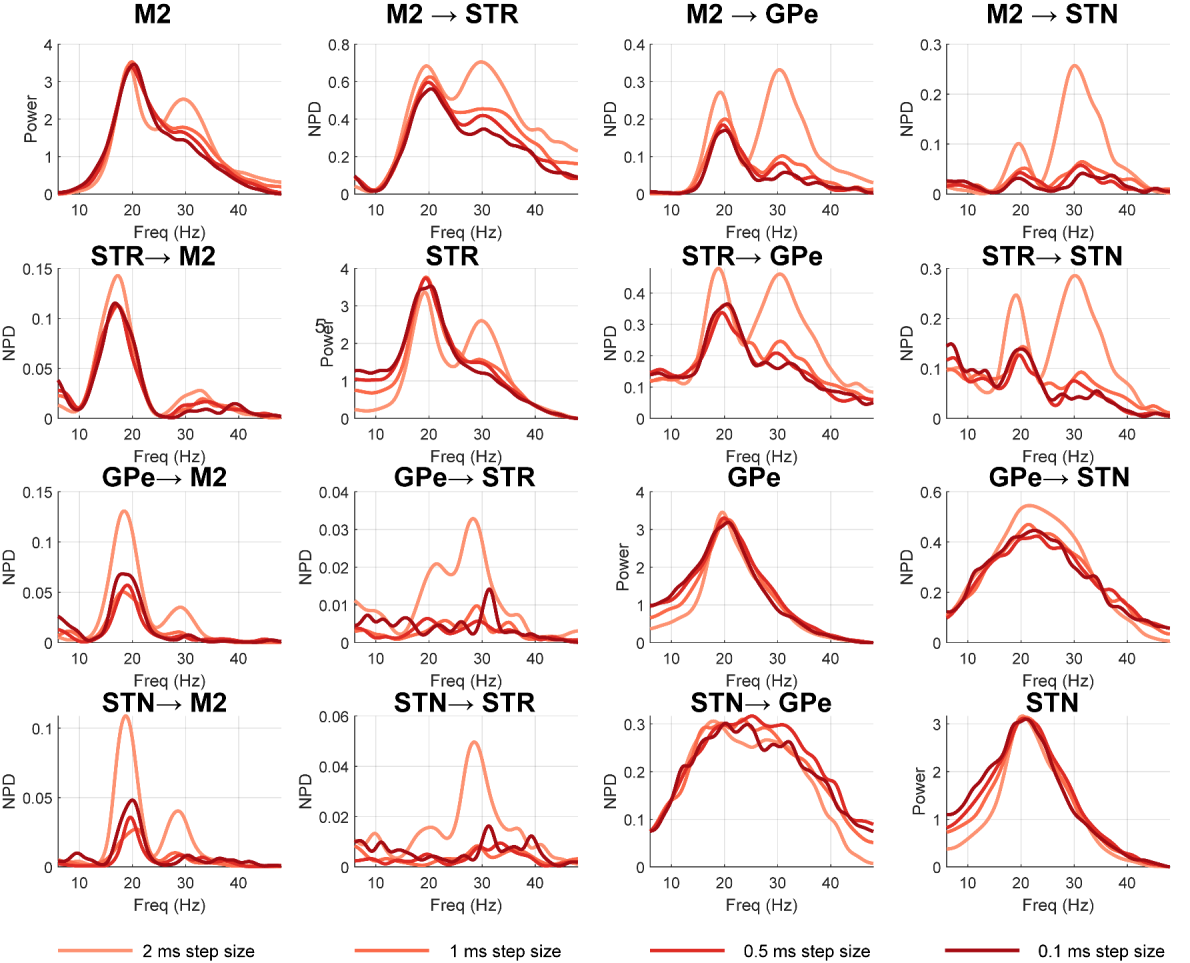


The posterior model 5.2 from figure 5 was simulated (256s) with MAP parameters but for different integration step-sizes. The Euler-Maruyama step size was varied from 2ms to 0.1ms. Simulations show that the numerical solutions are convergent with smaller step-sizes. Simulations in this paper use a 0.5ms step size, no qualitative change in the simulation summary statistics were found for a smaller step size at 0.1ms.

## Appendix III- Examination of forward uncertainty of posterior model


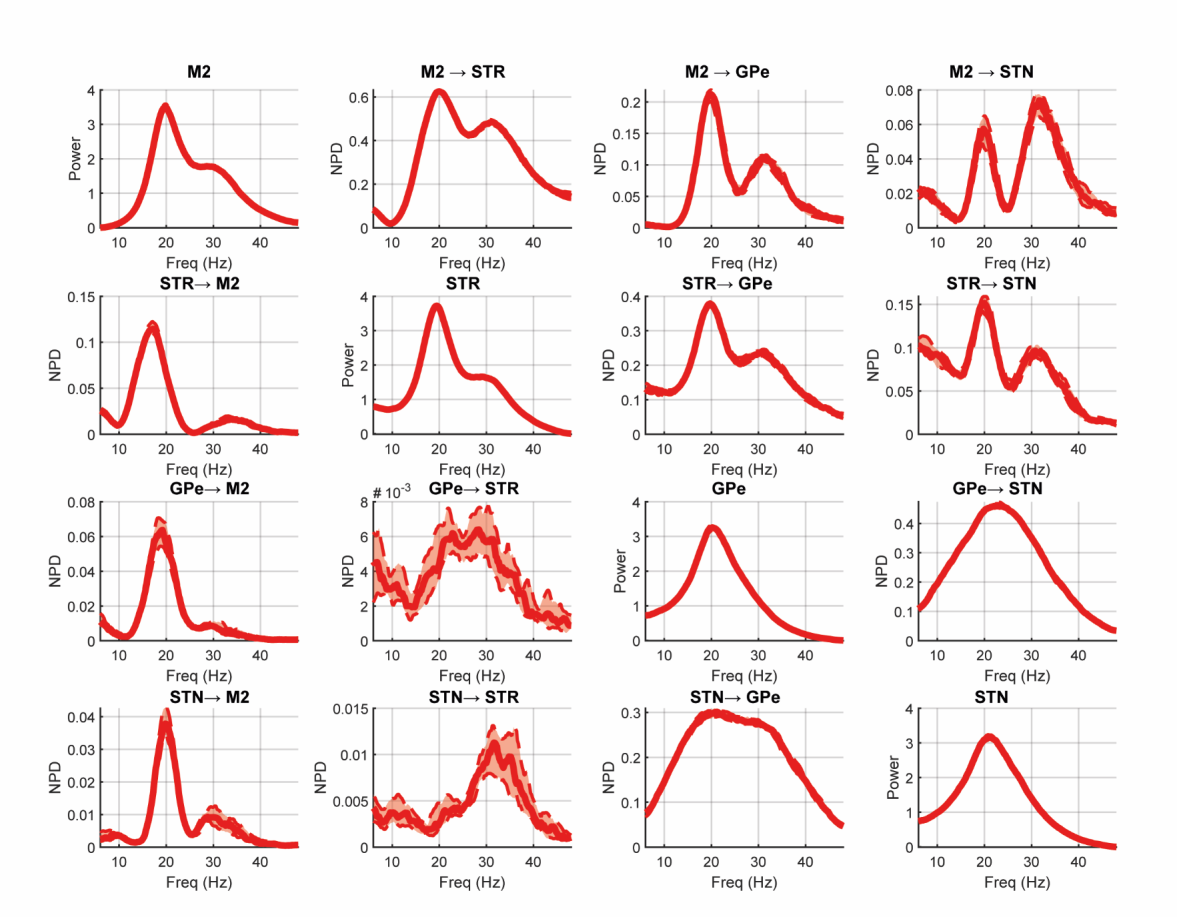


The posterior model 5.2 from figure 5 was repeatedly (n=25) simulated (256s) with MAP parameters but different realizations of the underlying noise process. This figure shows the averaged simulated data features alongside their interquartile range in the bounds. No major deviations were found across realizations, with all giving consistent spectral features.

## Appendix IV- Outline of Software and Scripts

We provide scripts to perform the analyses described in this paper in the open accessible GitHub repository. The script “West2021_Neuroimage_Figures.m” provides a master file linking to the respective computations required for each figure. Note that is some circumstances, the functions can be parallelized to run across multiple instances of MATLAB, running for instance on a distributed system (e.g. “Figure5_6_i_modelfitting.m”). These scripts use a shared Working List, called ‘WML.mat’, that must be accessible to all MATLAB instances.

The general structure of the software is given in the schematic below. The software can be broken down into (1) project specific files and (2) the generic procedures written to be applicable across a range of different datasets and models. Individual projects folders must include (A) a dataset – given as timeseries data, or data preprocessed and already transformed to the feature space of the summary statistics; (B) a set of model functions describing the equations of motion, and integration scheme that outputs a time series; (C) model priors specified in terms of their expectations and precisions; (D) a folder of model specifications outlining the parameterization of different models to be compared. All functions take a common configuration structure ‘R’ that sets various parameters and paths for the analyses. These are setup using “ABC_setup_*projectname*.m” that must be written for each new project.

Using the given template project, it should be possible to adapt the files to suit other sets of data and models. For bug-fixes, troubleshooting, or pull requests please see the GITHUB repository at: <https://github.com/twestWTCN/ABCNeuralModellingToolbox.git>.

**Schematic of software structure and key functions.** The software is split into two main branches: (A) project specific files designed for particular model and dataset; (B) generic procedures written to be broadly applicable across projects. Functions often use a configuration variable ‘R’ that created using “ABC_setup_....m” that defines various important parameters for the fitting and analysis.

1. These values indicate the variance of scaling constant *c*, where *c~N(μ,* σ^2^). Thus, the log scaled parameter *X* with mean value *x* is given by $X=xe^{c}$. [↑](#footnote-ref-1)
